# The efficacy-invasiveness trade-off: a retrospective cohort comparison of surgical operation and endobronchial valves for refractory lung bullae disease

**DOI:** 10.1186/s12890-026-04488-5

**Published:** 2026-07-10

**Authors:** Honghai Li, Zejin Zhang, Haoning Nan, yanbin Pei, Ming Zhao, Yafei Sun, Siyu Chen, Qiu Yonghui, Yuqi Wang

**Affiliations:** 1 Department of Thoracic Surgery, The First Medical Center, Chinese PLA General Hospital, Beijing, 100853 China; 2 Medical School of the Chinese General Hospital of PLA, Beijing, 100853 China; 3 Department of Thoracic Surgery, The Sixth Medical Center, Chinese PLA General Hospital, Beijing, 100089 China; 4 Department of Thoracic Surgery, The 304 Hospital of the PLA General Hospital, Beijing, 100048 China

**Keywords:** Refractory lung bullae disease, Retrospective cohort study, Surgical operation, Endobronchial valves

## Abstract

**Background:**

The optimal intervention for refractory bullae lung disease remains debated, with a choice between invasive surgical operation (SO) and the minimally invasive endobronchial valve (EBV). A comprehensive comparison of their long-term efficacy, safety, and cost is crucial for clinical decision-making.

**Objective:**

To compare perioperative outcomes, 1-year efficacy, long-term recurrence-free survival, and economic impact between surgical bullectomy (SO) and endobronchial valve (EBV) placement.

**Methods:**

In this comparative study, 673 patients with refractory bullae disease from two Chinese centers were enrolled (SO: *n* = 436; EBV: *n* = 237). This study enrolled patients with refractory bullous disease, defined by HRCT-confirmed bullae > 10 cm in diameter or concomitant symptoms impacting daily life, and an inadequate clinical response to a minimum of 3 months of optimized medical management. After applying propensity score matching (PSM) to control for confounding factors, perioperative outcomes, 1-year efficacy, and long-term recurrence‑free survival were compared between the two groups. Analyses employed analyses of covariance (ANCOVA), mixed-effects models, Kaplan-Meier estimates, Cox regression, and logistic regression.

**Results:**

Following PSM, excellent balance in baseline characteristics was achieved between the surgical (SO, *n* = 237) and endobronchial valve (EBV, *n* = 237) groups. Regarding perioperative outcomes, EBV was associated with a lower risk of complications (OR = 0.33), shorter operation time (mean difference: -0.72 h), less blood loss (mean: -48.00 ml), and a reduced hospital stay (mean: -7.61d) compared to SO (all *p* < 0.001). However, total hospitalization costs were significantly higher for EBV (mean increase: ¥104,502). For secondary outcomes, EBV demonstrated lower rates of radiologic lung re-expansion based on the semi-quantitative imaging assessment used in this study (OR = 0.15, *p* < 0.001) and bulla volume reduction (mean difference: -29.02%, *p* < 0.001) at 1 year, and was associated with a higher 1-year readmission risk (OR = 3.21, *p* < 0.001). For the primary outcomes at 1 year, SO demonstrated superior improvement in both lung function (adjusted mean difference in forced expiratory volume in one second (FEV1)%: -9.71%, *p* < 0.001) and exercise capacity (adjusted mean difference in 6-minute walk distance (6MWD): -53.04 m, *p* < 0.001). Mixed-effects models confirmed that SO’s advantages in FEV1% were significant at all time points, while its 6MWD benefit became fully apparent only by 1 year. Long-term recurrence-free survival was significantly higher in the SO group (2-year rate: 86.5% vs. 70.0%; HR for EBV: 2.48, *p* < 0.001). Subgroup analysis revealed this survival advantage was most pronounced for giant bullae (≥ 10 cm), where the recurrence risk with EBV was 3.49-fold higher (HR 3.49, *p* < 0.001), whereas for smaller bullae (< 10 cm) the risk was comparable between groups (HR 1.32, *p* = 0.426).

**Conclusion:**

SO was associated with greater improvements in pulmonary function, exercise capacity, and recurrence-free survival in this matched retrospective cohort. However, given the non-randomized design and the potential for residual confounding, these findings should be interpreted cautiously and require further validation in prospective studies. EBV offers a safer, minimally invasive alternative with faster recovery, but is limited by higher costs and an increased recurrence risk. The treatment choice should be individualized, strongly considering the patient’s chronic obstructive pulmonary disease (COPD) staging, tolerance for surgical risk, and valuation of long-term efficacy versus initial recovery and cost.

**Supplementary Information:**

The online version contains supplementary material available at https://doi.org/10.1186/s12890-026-04488-5.

## Introduction

Giant bullae pose a major clinical challenge by compressing functional lung tissue, resulting in severe dyspnea, recurrent infections, and impaired quality of life [1]. Additionally, multifocal or strategically located bullae can drive recurrent pneumothoraces or other symptoms refractory to medical management [2]. Although the treatment objectives, including symptom relief, functional improvement, and recurrence prevention, are well-defined, the optimal therapeutic strategy remains a subject of debate. Surgical operation (SO) [via thoracotomy or video-assisted thoracoscopic surgery (VATS)] remains the gold standard for eligible patients with adequate pulmonary reserve, as it reduces lung volume and facilitates re-expansion of healthy parenchyma [3]. However, its invasiveness and associated risks, including persistent air leaks and significant morbidity, limit its applicability in patients with advanced disease and comorbidities [4]. Minimally invasive bronchoscopic techniques have recently emerged as promising alternatives for achieving surgical-level physiological benefits with a lower risk of complications.

The endobronchial valve (EBV) technique, in particular, has gained traction. It functions by inducing lobar collapse through selective occlusion of the most diseased lung segments, leveraging the principle of regional lung volume reduction akin to surgery, yet via a less invasive pathway [5]. This aligns with the overarching shift in interventional pulmonology towards minimizing procedural trauma, accelerating recovery, and optimizing healthcare resource allocation [6]. Nonetheless, a direct comparison with surgical resection is imperative to substantiate these advantages and to definitively establish its relative efficacy, especially in terms of long-term outcomes, including durable functional improvement, exercise capacity, recurrence-free survival, and overall economic impact.

The assessment of any bullous lung disease treatment hinges critically on functional outcomes. FEV1% predicted serves as a pivotal indicator of airway obstruction and the immediate efficacy of decompression [7], while the 6MWD offers an integrated measure of functional exercise capacity relevant to quality of life [8]. A critical appraisal must therefore assess not only the magnitude of improvement in these parameters afforded by surgical and bronchoscopic techniques but also the longevity of benefit and long-term recurrence rates. Furthermore, procedural duration is a key differentiator. Extensive surgical bullectomy can entail prolonged operating times, elevating risks associated with anesthesia and surgical complexity, especially in compromised patients [9]. Bronchoscopic valve insertion, though technically nuanced, generally requires less time and can be performed under conscious sedation, potentially mitigating perioperative burden [10]. This advantage, however, must be weighed against the procedural learning curve and the dependency on specialized resources, which can diminish its applicability in resource-constrained environments .

Despite growing interest in bronchoscopic alternatives, the comparative effectiveness of SO and EBV for refractory bullae remains an area of significant uncertainty. A landmark study first established the feasibility of EBVs in inoperable patients, reporting sustained clinical benefits over 6 months [11]. This has been corroborated by numerous case reports (e.g., Hou et al., Lee et al.) detailing successful bulla reduction [12, 13]. Nevertheless, the current evidence is constrained by a preponderance of retrospective, single-arm investigations characterized by substantial clinical and methodological heterogeneity. Consequently, there is a critical imperative for well-controlled comparative analyses to generate definitive guidance.

This retrospective study is designed to address this unmet need by conducting a comparison of SO and EBV in a well-defined patient cohort. Our comprehensive evaluation encompasses key efficacy endpoints (FEV1% predicted, 6MWD, recurrence), procedural efficiency (operating time, length of stay), safety profiles, and cost-effectiveness. By providing this nuanced understanding, our work aims to directly shape evidence-based clinical pathways and resource allocation strategies. We anticipate that the findings will clarify the relative value of each modality, leading to more personalized treatment, enhanced procedural outcomes, and the equitable advancement of care for this challenging patient population.

## Materials and methods

### Sample size calculation

An a priori sample size calculation was performed using G Power software (version 3.1). The calculation was based on the primary outcome of this study, defined as the absolute change from baseline in the FEV1% pred. Based on previous literature [14, 15] and preliminary pilot data, we anticipated a mean improvement of 20% in the surgical bullectomy group and 12% in the endobronchial valve group, resulting in an expected mean difference of 8%. Assuming a pooled standard deviation (SD) of 12% for the change in FEV1% pred, the effect size (Cohen’s d) was calculated as 0.67. To detect this effect size with a two-sided significance level (α) of 0.05 and a power (1-β) of 80% using an independent two-sample t-test, a minimum of 36 participants per group (total *n* = 72) was required. Allowing for an estimated 15% dropout rate during the follow-up period, the final recruitment target was set at a minimum of 42 participants per group, for a total sample size of 84.

Commercially available one-way endobronchial valves (Zephyr^®^, Pulmonx Inc., USA) were used in this study. The number of valves deployed per patient varied according to individual bronchial anatomy and the extent of the target lobe, typically ranging from 2 to 5 valves. Target lobe selection was based on a comprehensive pre-procedural assessment integrating CT findings, including bulla location, emphysema distribution, and fissure completeness. In addition, the Chartis Pulmonary Assessment System was routinely used intraoperatively to assess collateral ventilation and confirm target lobe suitability. In cases where fissure integrity was clearly evident on CT, Chartis assessment was used to support decision-making but not as the sole determinant. Technical success of the procedure was defined as successful deployment of all planned valves with appropriate positioning and expansion, as confirmed by bronchoscopic visualization, and the absence of immediate major complications requiring procedural termination. Post-procedural management included routine monitoring for complications such as valve migration, mucus plugging, or airway obstruction. In cases of significant complications or inadequate clinical response, valve repositioning or removal was performed at the discretion of the treating physician. Valves were generally intended for long-term placement but could be electively removed if clinically indicated.

### Study population and patient selection

**Fig. 1:**
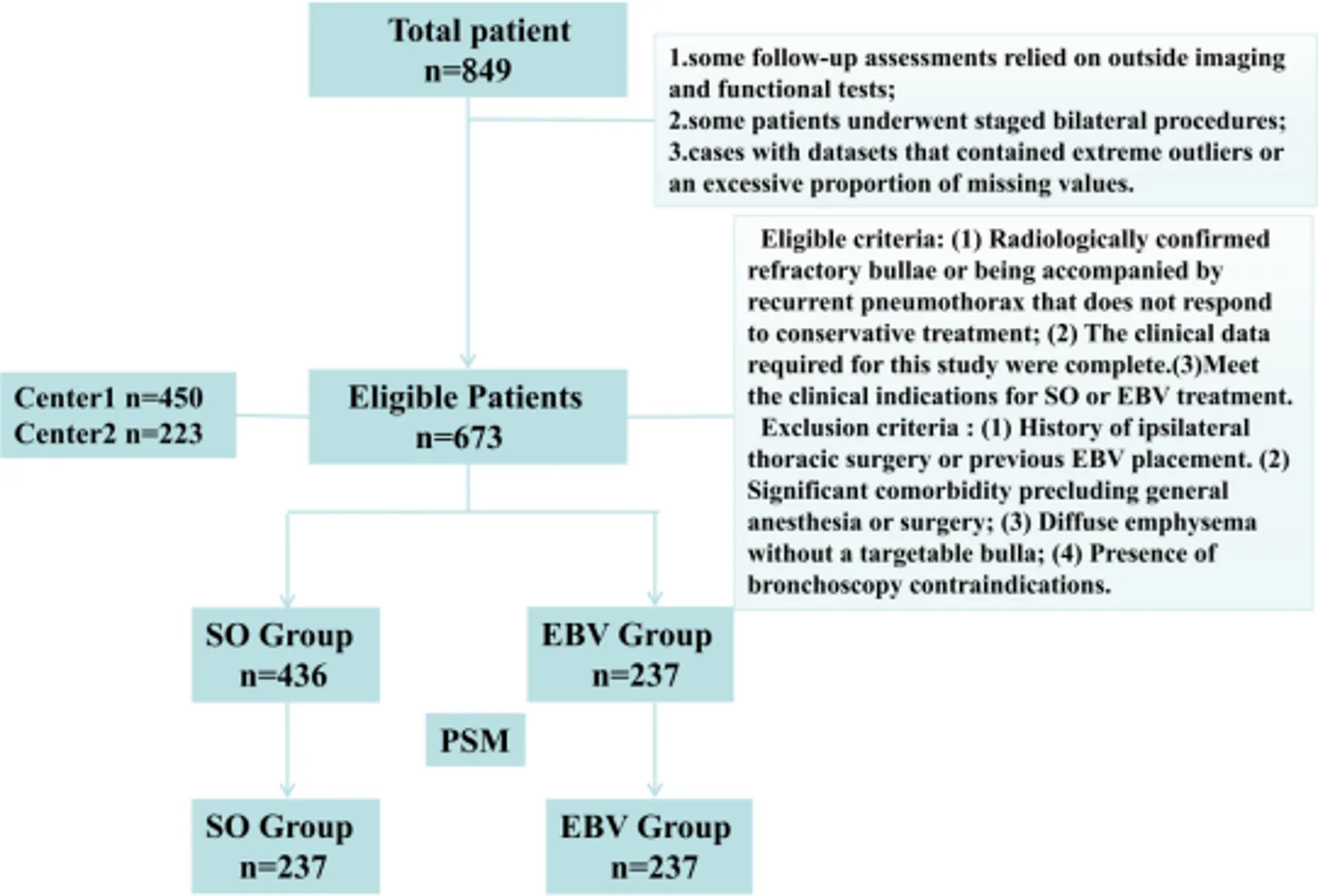
Flowchart of patient selection for the study cohort

This study was a comparative analysis of retrospectively collected data from 450 patients (SO: *n* = 287; EBV: *n* = 163) treated at Center 1, and 223 patients (SO: *n* = 149; EBV: *n* = 74) treated at Center 2, yielding a total of 673 patients with refractory bullous disease between January 2015 and December 2023 (Fig. 1). As this was a cohort study, the ethics committee waived the requirement for written informed consent. In addition, the study complied with all applicable guidelines and regulations.

Patients were eligible if they met the following criteria: (1) Radiologically confirmed refractory bullae (defined as having a maximum bulla diameter exceeding 10 cm) on high-resolution computed tomography (HRCT) or concomitant symptoms impacting daily life, and an inadequate clinical response to a minimum of 3 months of optimized medical management; (2) The clinical data required for this study were complete, and the patients returned to our outpatient clinic for follow-up reviews at 1 month, 6 months, 1 year, and 2 years after discharge as medically advised. (3) Meet the clinical indications for SO or EBV treatment. Key exclusion criteria were: (1) History of ipsilateral thoracic surgery or previous EBV placement. (2) Significant comorbidity precluding general anesthesia or surgery (e.g. severe heart failure, uncontrolled pulmonary hypertension); (3) Diffuse emphysema without a targetable bulla; (4) Presence of bronchoscopy contraindications (such as airway malformation, risk of severe hemoptysis, etc.). Optimized medical management included standard guideline-directed therapy for underlying chronic obstructive pulmonary disease (COPD) or bullous lung disease, comprising bronchodilator therapy (long-acting β2-agonists and/or anticholinergics), inhaled corticosteroids when indicated, smoking cessation counseling, pulmonary rehabilitation, and management of respiratory infections or exacerbations as appropriate.

In the surgical group, pleural abrasion was routinely performed following bullectomy to promote pleurodesis and reduce the risk of postoperative pneumothorax recurrence. Postoperative management was conducted according to standardized institutional protocols. Chest tubes were connected to a standard water-seal drainage system with low-pressure suction when clinically indicated. When suction was applied, a negative pressure of approximately − 10 to − 20 cmH₂O was maintained according to institutional practice and the patient’s postoperative air leak status. Chest tube drainage characteristics were routinely monitored, and the fluid was typically serous or mildly serosanguineous at the time of removal. Chest tube removal was considered when there was no evidence of ongoing air leak, satisfactory lung re-expansion was confirmed on chest radiography, and the daily drainage volume was less than 200 mL. The threshold of 200 mL/day was selected based on commonly accepted thoracic surgical practice and previous evidence suggesting that chest tube removal at this drainage level is safe and does not increase the risk of pleural effusion-related complications [21]. Postoperative analgesia was provided using a multimodal approach, including non-opioid analgesics and, when necessary, patient-controlled analgesia (PCA) with opioids. Discharge criteria included stable vital signs, adequate pain control with oral medications, satisfactory lung re-expansion on chest imaging, and the absence of active complications. All procedures were performed by experienced thoracic surgeons at each participating center. Although minor variations in surgical technique and perioperative management may have existed between centers, all teams followed broadly comparable clinical practice standards.

### Treatment allocation process

Treatment allocation was not randomized but was determined through a multidisciplinary clinical decision-making process. Specifically, all cases were jointly evaluated by a team consisting of thoracic surgeons and pulmonologists, with input from the patient after discussion of risks and expected benefits. In general, surgical bullectomy (SO) was considered the first-line treatment for patients with adequate pulmonary reserve, acceptable operative risk, and anatomically resectable bullae. In contrast, endobronchial valve (EBV) placement was preferentially considered for patients who were deemed less suitable for surgery due to relatively higher procedural risk, borderline pulmonary function, or patient preference for a less invasive approach. Although formal institutional criteria were not strictly protocolized across the entire study period, the decision-making process consistently incorporated key clinical parameters, including COPD severity (Global Initiative for Chronic Obstructive Lung Disease (GOLD) stage), baseline pulmonary function (FEV1% predicted), distribution and size of bullae, and overall functional status. It should be noted that patients with severe comorbidities absolutely precluding general anesthesia or bronchoscopy were excluded from both groups, resulting in a study population representing patients who were technically eligible for either intervention but differed in relative risk profiles and clinical considerations.

### Treatment modalities

**Fig. 2:**
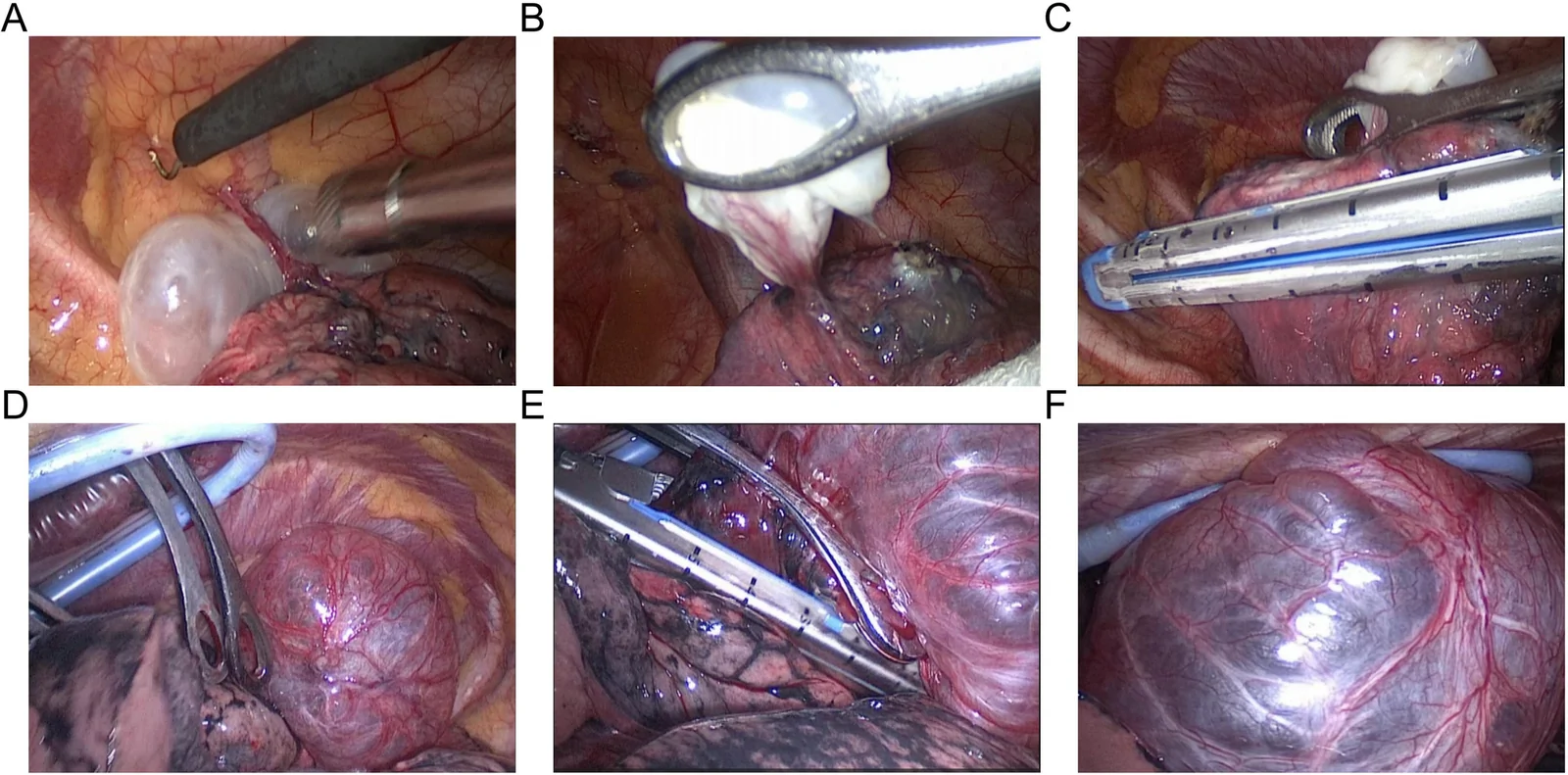
The surgical procedures for the two patients (Cases **A**–**C** and **D**–**E**) are delineated sequentially. In both cases, the initial step involved adequate exposure and blunt dissection to visualize the pulmonary bulla and establish a sufficient working space (Step **A**). Subsequently, the bulla was grasped and elevated to expose its pedicle (Steps **B** and **D**), followed by resection using a linear cutting stapler (Steps **C** and **E**). Finally, the resected specimens were retrieved through the working port (Step **F**)

Patients in the SO group underwent VATS under general anesthesia with double-lumen endotracheal intubation, positioned in the lateral decubitus position with the non-operative side dependent. A standardized two-port approach was employed. An initial 1–1.5 cm incision was made at the 7th or 8th intercostal space along the mid-axillary line for the introduction of a 30° thoracoscope, serving as the observational port. This allowed for a comprehensive assessment of intrapleural adhesions and the precise location, size, and number of bullae. In cases of pleural adhesions, sharp dissection was performed using an ultrasonic scalpel and electrocautery. A second 3–4 cm utility port was established at the 4th intercostal space along the anterior axillary line. The lung parenchyma was systematically inspected using a non-crushing oval clamp. Bullae and bullous tissues were grasped with the oval clamp and resected at their base using an endoscopic stapling device (Fig. 2). In the majority of surgical cases, resection was performed using an Endo GIA stapling device (Medtronic, USA). Depending on tissue thickness and anatomical characteristics, vascular or standard lung tissue cartridges were selected at the discretion of the operating surgeon. Among the patients undergoing surgical treatment, 218 patients underwent stapled bullectomy using Endo GIA devices. Staple-line reinforcement with titanium clips was required in 42 patients because of minor air leakage detected during intraoperative underwater testing, whereas suture ligation was performed in 17 patients with pedunculated bullae or when additional reinforcement was considered necessary. For scattered small bullae or staple lines demonstrating air leakage, reinforcement was achieved with dual titanium clip application, with or without preceding bipolar electrocautery. Pedunculated bullae were managed by grasping the pedicle and securing its base with non-absorbable suture ligation. Upon completion of either resection or ligation, an underwater air leak test was routinely performed to ensure parenchymal integrity. Pleural abrasion of the parietal pleura was then conducted to promote pleurodesis and mitigate the risk of postoperative pneumothorax recurrence. A single 28 F chest tube was inserted through the 7th or 8th intercostal space at the mid-axillary line, with the side port positioned approximately 3 cm from the pleural surface. For a subset of patients with giant bullae occupying the majority of the hemithorax, resulting in severe compression of normal lung tissue and obscuration of anatomical planes, or for bullae with an exceptionally broad base rendering safe and complete stapler resection unfeasible, an open thoracotomy approach was utilized. The patient positioning and fundamental resection principles remained consistent with the VATS approach. A posterolateral thoracotomy incision was performed, starting between the anterior axillary and midclavicular lines at the 5th intercostal space and extending posteriorly to the region between the spine and scapula.

**Fig. 3:**
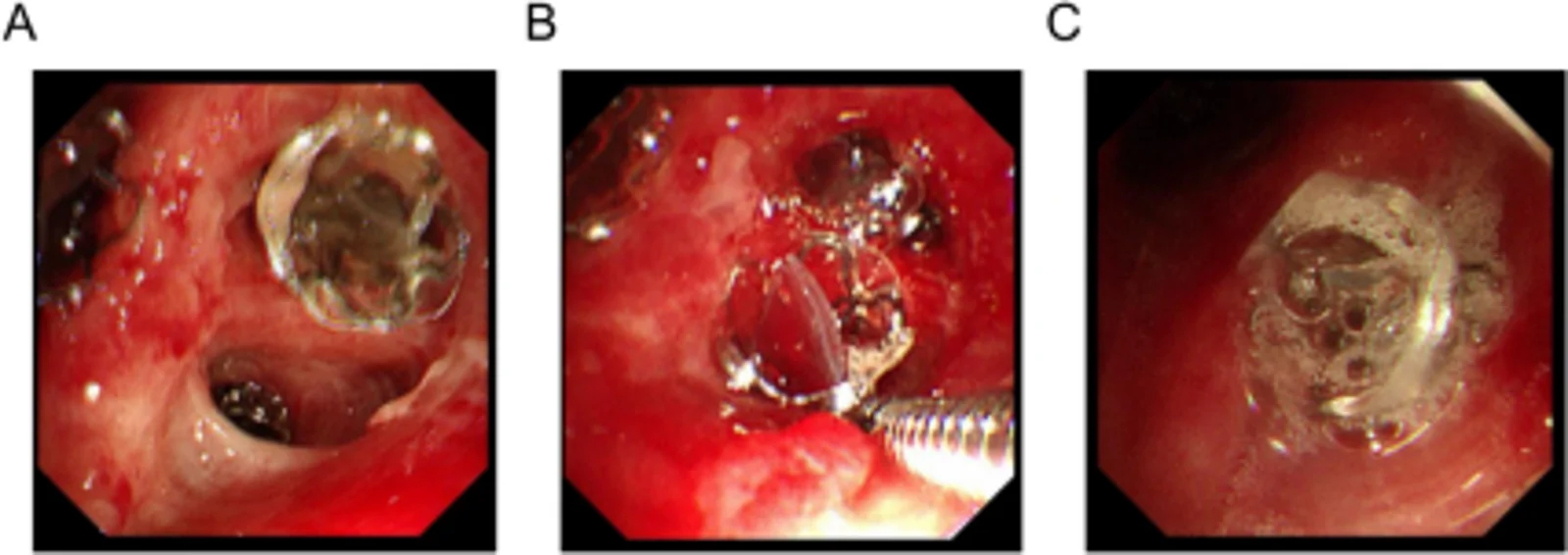
Schematic illustration of endobronchial valve implantation. **a**, **b** demonstrate the positioning of a unidirectional endobronchial valve in the anterior segment of the right upper lobe from different perspectives, respectively. **c** shows valve placement within the lingular segment of the left upper lobe

The EBV group procedure was performed under general anesthesia with patients in the supine position. Prior to valve deployment, the Chartis Pulmonary Assessment System (Pulmonx Inc., Redwood City, CA, USA) was utilized to objectively evaluate the presence or absence of collateral ventilation to the target lobe. Based on the bronchoscopic anatomy and collateral ventilation status, one or more one-way endobronchial valves were deployed into the segmental bronchi supplying the target bullous lobe via the working channel of a flexible bronchoscope(Figure 3). Post-deployment, bronchoscopic visualization was used to confirm proper valve expansion and optimal positioning.

Within the surgical operation (SO) group, the majority of patients underwent video-assisted thoracoscopic surgery (VATS), while open thoracotomy was reserved for a subset of patients with giant bullae occupying a large hemithorax, complex anatomical relationships, or when safe stapled resection could not be achieved thoracoscopically. Due to the retrospective nature of the study and variations in surgical documentation across centers and time periods, detailed stratification of surgical approaches (VATS versus thoracotomy) was not consistently available for all patients and therefore was not included as a matching variable or subgroup analysis factor.

Among the 237 patients in the surgical group after propensity score matching, 214 patients (90.3%) underwent video-assisted thoracoscopic surgery (VATS), whereas 23 patients (9.7%) required open thoracotomy. All patients treated via thoracotomy presented with giant bullae measuring ≥ 10 cm in maximum diameter and exhibited either severe compression of the remaining lung parenchyma, obscured anatomical planes, or broad-based bullae that were considered unsuitable for safe thoracoscopic resection.

### Clinical data

Clinical data were retrospectively collected from the enrolled patients for subsequent analyses. The study variables were categorized into four domains.Baseline demographic and clinical characteristics included age, sex, smoking history, history of occupational dust exposure, history of asthma, and history of underlying diseases. The latter represented a composite variable including common chronic conditions such as hypertension, diabetes mellitus, and cardiovascular diseases. Disease-specific characteristics included COPD staging according to the GOLD criteria, number of pulmonary bullae, maximum diameter of pulmonary bullae, anatomical location of bullae, and classification of pulmonary bullae based on radiologic features. Preoperative physiological parameters included forced expiratory volume in one second (FEV1, L), FEV1% predicted, forced vital capacity (forced vital capacity (FVC), L), FVC% predicted, and 6-minute walk distance (6MWD, m). Perioperative variables included total operation time (hours), intraoperative blood loss (mL), postoperative hospital stay (days), perioperative complications, and total hospitalization cost. Postoperative efficacy outcomes included FEV1% predicted and 6MWD measured at 1 month, 6 months, and 1 year after the procedure. Radiologic outcomes included the reduction rate of bulla size on CT at 1 year and re-expansion of adjacent lung tissue. Clinical outcomes further included all-cause readmission within 1 year and recurrence status, both of which were defined as described below. All-cause readmission was defined as any unplanned hospital admission occurring within 1 year after the index procedure. Although the primary causes of readmission were predominantly related to respiratory conditions, including pneumothorax recurrence, infection, or symptom exacerbation, cause-specific readmission data were not consistently available for all patients. Pulmonary bullae were categorized into three radiologic patterns based on preoperative high-resolution computed tomography (HRCT) findings, adapted from previously described classifications of bullous emphysema and giant bullous disease. Type I was defined as an isolated giant bulla with relatively preserved surrounding lung parenchyma. Type II referred to multiple bullae with partial preservation of adjacent lung tissue. Type III represented diffuse or widespread bullous disease with extensive background emphysematous destruction [16]. Classification was determined independently by two experienced radiologists based on HRCT imaging, with discrepancies resolved by consensus. This categorization was used to characterize disease morphology and severity in the present study.

**Fig. 4:**
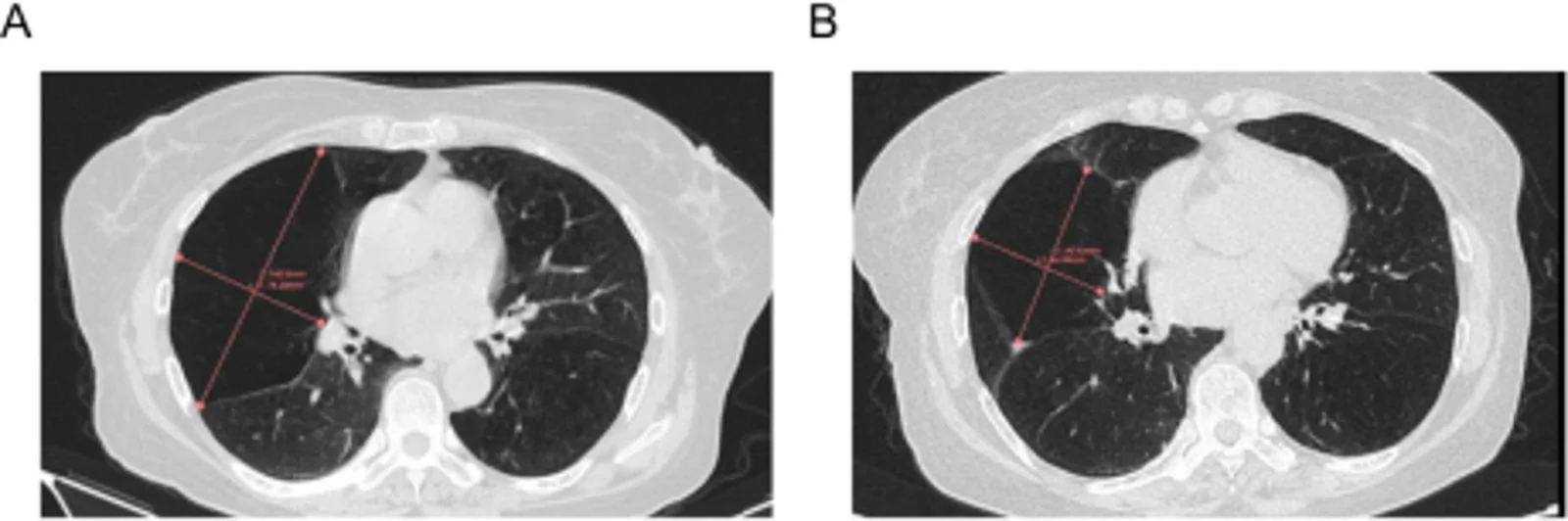
Quantitative assessment of bulla volume. Representative imaging shows the bulla (**a**) before and (**b**) after endobronchial valve implantation, demonstrating volume reduction

Preoperative and postoperatively CT images were collected from patients. Specifically, the spiral scanning mode was utilized with a tube voltage set at 120 kVp and the effective tube current maintained between 200 and 250 mAs. The acquisition matrix was 512 × 512, the field of view was 350 × 350 mm, and the detector collimation was 16 × 1.5 mm. The rotation time was 0.75 s, with a slice thickness and slice interval both of 1 mm. The slice thickness for CT reconstruction was 1 mm. All images were exported in the Digital Imaging and Communications in Medicine (DICOM) format. The reduction rate of the target bulla was quantitatively assessed on CT scans. At both baseline and the 1-year, the axial slice demonstrating the largest cross-sectional area of the bulla was identified. The longest diameter (L1) and the perpendicular short diameter (L2) of the bulla were measured on this slice. The product of these two diameters (L1 × L2) was calculated, and the percentage reduction in this product was used as a surrogate for volumetric change (Fig. 4). And re-expansion of adjacent lung tissue was evaluated using a semi-quantitative visual scoring system on CT scans, and radiologic re-expansion of the adjacent compressed lung tissue was defined as restoration of more than 50% of the previously compressed lung parenchymal volume, as assessed by comparative CT imaging. All scans were reviewed independently by two experienced radiologists who were blinded to the clinical data. In cases of disagreement, a consensus was reached through joint review and discussion. Due to the retrospective nature of the study, formal inter-observer agreement analysis (e.g., kappa statistics or intraclass correlation coefficients) was not consistently performed; however, all measurements were finalized based on consensus to ensure reliability. Re-expansion of adjacent lung tissue was assessed using a semi-quantitative visual scoring method based on CT imaging, defined as greater than 50% restoration of compressed lung volume. This assessment was performed independently by two experienced radiologists, with discrepancies resolved by consensus. However, this scoring approach has not been formally validated and should be interpreted as an approximate indicator of lung re-expansion. Bulla volume reduction was estimated using a two-dimensional surrogate measurement (L1 × L2) on the axial slice demonstrating the largest cross-sectional area of the bulla. Due to the retrospective nature of the study, standardized three-dimensional volumetric analysis was not consistently available. Therefore, this method was used as a practical approximation of volumetric change. Recurrence was defined as the occurrence of any of the following events during follow-up: (1) radiologic reappearance or progression of bullae on chest CT; (2) recurrence of pneumothorax confirmed by imaging; or (3) the need for reintervention (surgical or bronchoscopic) due to recurrent bullous disease. Symptom recurrence alone without radiologic or clinical confirmation was not considered sufficient to define recurrence. The time to recurrence was calculated from the date of the initial procedure to the first occurrence of any of the above events.

Due to the retrospective nature of this study and the long inclusion period across two centers, several clinically relevant covariates were not consistently available in the medical records and therefore could not be reliably extracted for all patients. These variables included detailed comorbidity burden (e.g., Charlson Comorbidity Index), frailty or functional status assessments, diffusion capacity of the lung for carbon monoxide (DLCO), quantitative assessment of emphysema severity and distribution, prior history of pneumothorax or interventional procedures, and nutritional indicators such as body mass index. In addition, although collateral ventilation status was assessed using the Chartis system in patients undergoing EBV placement, this information was not available for patients in the surgical group and was therefore not included as a baseline covariate for comparative analysis. The variable “history of underlying diseases” in this study represents a composite indicator including common chronic conditions such as hypertension, diabetes mellitus, and cardiovascular diseases; however, detailed stratification of specific comorbidities was not uniformly documented and thus could not be further analyzed.

### Statistical analysis

Ontinuous variables with a normal distribution were described as mean ± standard deviation and compared between groups using Welch’s t-test. Continuous covariates with a non-normal distribution were expressed as median (interquartile range) and compared using the Wilcoxon rank-sum test. Categorical covariates were presented as counts and percentages and compared using the chi-square test or Fisher’s exact test when cell counts were small. PSM was employed to balance covariates between treatment groups and mitigate the effects of potential confounding factors. We performed 1:1 optimal pair matching. The matching was optimal in the sense that the sum of the absolute pairwise distances in the matched sample was as small as possible. The method functionally relied on the R package MatchIt. Analysis of covariance (ANCOVA) was employed to compare continuous outcomes. When baseline values were available, they were adjusted. Results are reported as least-squares (LS) means with 95% confidence intervals (CIs). For binary outcomes, logistic regression was used, with results expressed as odds ratios (ORs) and 95% CIs. A mixed-effects model was applied to analyze between-group differences and trends over time for repeatedly measured postoperative outcomes. Kaplan–Meier curves were generated to visualize recurrence-free survival, and between-group comparisons were performed using the log-rank test. The Cox proportional hazards model was used to estimate hazard ratios (HRs) with 95% CIs. Pre-specified subgroup analyses were conducted based on gender, smoking history, COPD stage, number of bullae, and maximum bulla diameter. These subgroup variables were selected a priori based on their established clinical relevance to disease severity, pulmonary functional reserve, and their potential influence on treatment selection and clinical outcomes in patients with bullous lung disease and emphysema-related lung volume reduction interventions [17, 18]. Interaction terms between treatment and subgroup factors were tested. A two-sided *p*-value < 0.05 was considered statistically significant. All statistical analyses were performed using R version 4.2.2 and SPSS Statistics 27.0.

Perioperative complications were defined as any adverse events occurring within 30 days after the procedure or during the index hospitalization. These included, but were not limited to, prolonged air leak (> 5 days), postoperative pneumothorax, hemothorax, pulmonary infection, respiratory failure requiring ventilatory support, and significant bleeding requiring intervention. Due to the retrospective design, complications were identified based on clinical documentation in the medical records, and a standardized grading system such as the Clavien-Dindo classification was not consistently applicable across all cases.

## Results

### Baseline characteristics

Table 1 presents the balance of baseline characteristics in the internal medicine and surgical treatment groups before and after propensity score matching. Each row corresponds to a specific covariate, with columns displaying the mean or proportion for each group, along with the standardized mean differences (SMDs) and their corresponding p-values before and after matching. Post-matching SMDs below 0.1 for all covariates confirmed that propensity score matching successfully achieved an excellent balance between the treatment groups. Many covariates that previously showed *p*-values < 0.05 now display *p*-values > 0.05. This change in p-values confirms that our propensity score matching approach successfully balanced the distribution of baseline characteristics between the groups. The improved balance enhances the reliability of treatment effect comparisons by minimizing the potential influence of confounding factors, thereby strengthening the credibility of our findings and their applicability to clinical practice. Pulmonary bullae were classified into Types I–III according to the predefined radiological classification described in the Methods section. Within the matched surgical cohort, VATS was the predominant surgical approach, accounting for 214 cases (90.3%), while 23 patients (9.7%) underwent open thoracotomy. Notably, all thoracotomy procedures were performed in patients with giant bullae (≥ 10 cm).

**Table 1:** Baseline characteristics of patients before and after propensity score matching

Covariates	Before Matching				After Matching			
	SO(*n* = 436)	EBV(*n* = 237)	SMD^△^	*P*	SO(*n* = 237)	EBV(*n* = 237)	SMD^△^	*P*
Age (mean (SD))	50.53 (10.47)	50.61 (9.82)	0.008	0.920	50.61 (10.01)	50.61 (9.82)	0.000	0.996
Preoperative.FEV1.L. (mean (SD))	1.82 (0.47)	1.85 (0.68)	0.039	0.589	1.85 (0.50)	1.85 (0.68)	0.006	0.941
Preoperative.FEV1.Predicted (mean (SD))	59.54 (15.63)	62.45 (20.34)	0.143	0.056	61.64 (16.57)	62.45 (20.34)	0.040	0.636
Preoperative.FVC.L. (mean (SD))	3.13 (0.67)	2.99 (0.93)	-0.151	0.041	3.01 (0.70)	2.99 (0.93)	-0.025	0.757
Preoperative.FVC.Predicted (mean (SD))	80.98 (13.48)	77.28 (17.92)	-0.206	0.006	78.21 (15.14)	77.28 (17.92)	-0.051	0.545
Preoperative.6.Minute.Walk.Distance.m. (mean (SD))	359.31 (68.23)	340.02 (88.24)	-0.219	0.004	344.25 (67.66)	340.02 (88.24)	-0.048	0.558
Gender (%)				0.927				> 0.999
Male	339 (77.8)	185 (78.1)	0.007		185 (78.1)	185 (78.1)	0.000	
Female	97 (22.2)	52 (21.9)	-0.007		52 (21.9)	52 (21.9)	0.000	
Smoking.history (%)				0.087				0.810
No	104 (23.9)	43 (18.1)	-0.148		41 (17.3)	43 (18.1)	0.022	
Yes	332 (76.1)	194 (81.9)	0.148		196 (82.7)	194 (81.9)	-0.022	
History.of.occupational.dust.exposure (%)				0.249				0.686
No	433 (99.3)	233 (98.3)	-0.078		235 (99.2)	233 (98.3)	-0.066	
Yes	3 (0.7)	4 (1.7)	0.078		2 (0.8)	4 (1.7)	0.066	
History.of.asthma (%)				0.307				0.510
No	382 (87.6)	201 (84.8)	-0.078		206 (86.9)	201 (84.8)	-0.059	
Yes	54 (12.4)	36 (15.2)	0.078		31 (13.1)	36 (15.2)	0.059	
History.of.underlying.diseases (%)				0.952				> 0.999
No	321 (73.6)	175 (73.8)	0.005		175 (73.8)	175 (73.8)	0.000	
Yes	115 (26.4)	62 (26.2)	-0.005		62 (26.2)	62 (26.2)	0.000	
COPD.Staging.by.GOLD.Criteria. (%)				0.226				0.456
˂3	340 (78.0)	175 (73.8)	-0.094		182 (76.8)	175 (73.8)	-0.067	
≥ 3	96 (22.0)	62 (26.2)	0.094		55 (23.2)	62 (26.2)	0.067	
Classification.of.Pulmonary.Bullae.Characteristics (%)				0.764				0.974
I	80 (18.3)	49 (20.7)	0.057		47 (19.8)	49 (20.7)	0.021	
II	343 (78.7)	181 (76.4)	-0.054		183 (77.2)	181 (76.4)	-0.020	
III	13 (3.0)	7 (3.0)	-0.002		7 (3.0)	7 (3.0)	0.000	
Number.of.Pulmonary.Bullae (%)				0.635				> 0.999
˂3	170 (39.0)	88 (37.1)	-0.038		88 (37.1)	88 (37.1)	0.000	
≥ 3	266 (61.0)	149 (62.9)	0.038		149 (62.9)	149 (62.9)	0.000	
Maximum.Diameter.of.Pulmonary.Bullae (%)				0.092				0.686
˂10	151 (34.6)	67 (28.3)	-0.141		71 (30.0)	67 (28.3)	-0.037	
≥ 10	285 (65.4)	170 (71.7)	0.141		166 (70.0)	170 (71.7)	0.037	
Location.of.Pulmonary.Bullae (%)				0.581				0.870
Left Upper Lobe	185 (42.4)	95 (40.1)	-0.048		106 (44.7)	95 (40.1)	-0.095	
Right Lower Lobe	46 (10.6)	20 (8.4)	-0.076		20 (8.4)	20 (8.4)	0.000	
Right Upper Lobe	159 (36.5)	92 (38.8)	0.048		83 (35.0)	92 (38.8)	0.078	
Right Middle Lobe	24 (5.5)	12 (5.1)	-0.020		10 (4.2)	12 (5.1)	0.038	
Left Lower Lobe	22 (5.0)	18 (7.6)	0.096		18 (7.6)	18 (7.6)	0.000	

*Abbreviations*: *COPD* Chronic obstructive pulmonary disease, *FEV1* Forced expiratory volume in 1 s, *FVC* Forced vital capacity, *6MWD* 6-minute walk distance, *SMD* Standardized mean difference

Bullae were classified into three types based on size, distribution, and anatomical characteristics: Type I: Single giant bulla with clear boundary, occupying a single lobe, with minimal surrounding emphysema.Type II: Multiple bullae involving one or more lobes, with moderate surrounding emphysema.Type III: Diffuse bullae throughout the lung parenchyma with severe background emphysema. *Abbreviations*: *SD* Standard deviation, *SMD* Standardized mean difference, *COPD* Chronic obstructive pulmonary disease, *FEV1* Forced expiratory volume in 1 s, *FVC* Forced vital capacity, *6MWD* 6-minute walk distance

^△^Standardized Mean Difference

### Comparison of perioperative metrics

We compared the perioperative outcomes between the two groups after propensity score matching, with the primary endpoints comprising perioperative complications, total operation time, total intraoperative blood loss, postoperative hospital stay, and total hospitalization costs. Perioperative complications were defined as adverse events occurring within 30 days after the procedure or during the index hospitalization. These included prolonged air leak (> 5 days), pneumothorax, hemothorax, pulmonary infection, respiratory failure requiring ventilatory support, and significant bleeding requiring intervention. In the EBV group, valve-related complications such as valve migration and airway obstruction were also recorded.

As shown in Table 2, compared with the SO group, the EBV group had a significantly lower risk of perioperative complications (OR = 0.33, *p* < 0.05). As shown in Table 3, ANCOVA was performed for total operation time, total intraoperative blood loss, postoperative hospital stay, and total hospitalization cost. The results indicated statistically significant differences between the EBV group and the SO group in all metrics (*p* < 0.001). Regarding operative characteristics, EBV had a significantly shorter operation time than SO, with a mean reduction of 0.72 h (95%CI:-0.80, -0.64). In terms of intraoperative blood loss, the EBV resulted in significantly less bleeding, showing a mean decrease of 48.00 ml compared to SO (95%CI:-54.11,-41.90). For postoperative recovery, patients undergoing EBV had a significantly shorter postoperative hospital stay than those receiving surgical operation, with a mean reduction of 7.61 days (95%CI:-8.33,-6.88). From an economic perspective, the total hospitalization cost for EBV was significantly higher than that for SO, with a mean increase of Renminbi (RMB) 104,502.21 (95%CI: 99,719.03 ,109,285.39). The detailed distribution of perioperative complications is presented in Table 2. Among patients undergoing surgical operation (SO), prolonged air leak (> 5 days) was the most common postoperative complication. A total of 27 patients (11.4%) developed persistent air leak, of whom most were managed conservatively with continued chest drainage and observation. Only three patients required additional interventions, and no procedure-related mortality was observed. In the EBV group, persistent air leak attributable to the procedure was uncommon, occurring in 2 patients (0.8%).

**Table 2:** Detailed comparison of perioperative complications between the two treatment approaches

Complication Type	Surgical Operation (*n* = 237)	Endobronchial Valve (*n* = 237)	*p*-value
Persistent air leak (> 5 days)	28 (11.8%)	2 (0.8%)	< 0.001
Pneumothorax	5 (2.1%)	3 (1.3%)	0.472
Pulmonary infection	4 (1.7%)	2 (0.8%)	0.412
Hemothorax	2 (0.8%)	—	—
Respiratory failure	2 (0.8%)	—	—
Significant bleeding	2 (0.8%)	—	—
Valve migration	—	2 (0.8%)	—
Airway obstruction (mucus plug)	—	2 (0.8%)	—
Total patients with complications	43 (18.0%)	11 (4.5%)	< 0.001

*Abbreviations*: *CI* Confidence Interval, *OR* Odds Ratio

**Table 3:** Comparison of Perioperative metrics Between the Two Treatment Modalities

Character	Mean (SD)	Difference in LS Mean (95% CI)^a^	*p*-value
Total.operation.time. (h)			
Endobronchial valve -Surgical operation	0.8 ( 0.26) -1.5 ( 0.54)	-0.72 (-0.80, -0.64)	< 0.001
Total.Intraoperative.Blood.Loss. (ml)			
Endobronchial valve -Surgical operation	1.4 ( 1.82) − 49.4 (47.94)	-48.00 (-54.11, -41.90)	< 0.001
Postoperative.Hospital.Stay.(d).			
Endobronchial valve -Surgical operation	3.1 ( 2.08) -10.7 ( 5.31)	-7.61 (-8.33, -6.88)	< 0.001
Total.Hospitalization.Cost.(RMB)			
Endobronchial valve -Surgical operation	125825.9 (36252.80)- 21323.7 (9858.93)	104502.21 (99719.03, 109285.39)	< 0.001

^**a**^Based on an ANOVA model

*ANCOVA* Analysis of Covariance, *CI* Confidence Interval, *LS* Least Squares, *SD* Standard Deviation

**Fig. 5:**
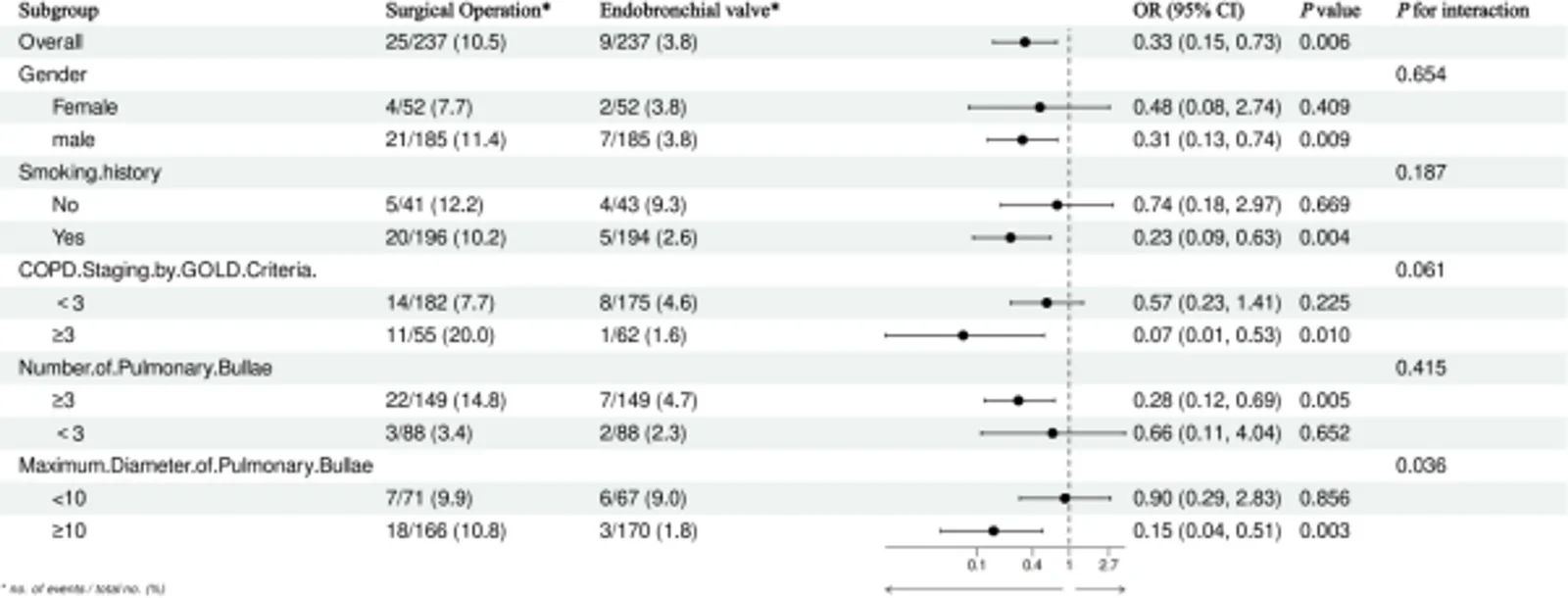
Subgroup analyses of perioperative complications

**Fig. 6:**
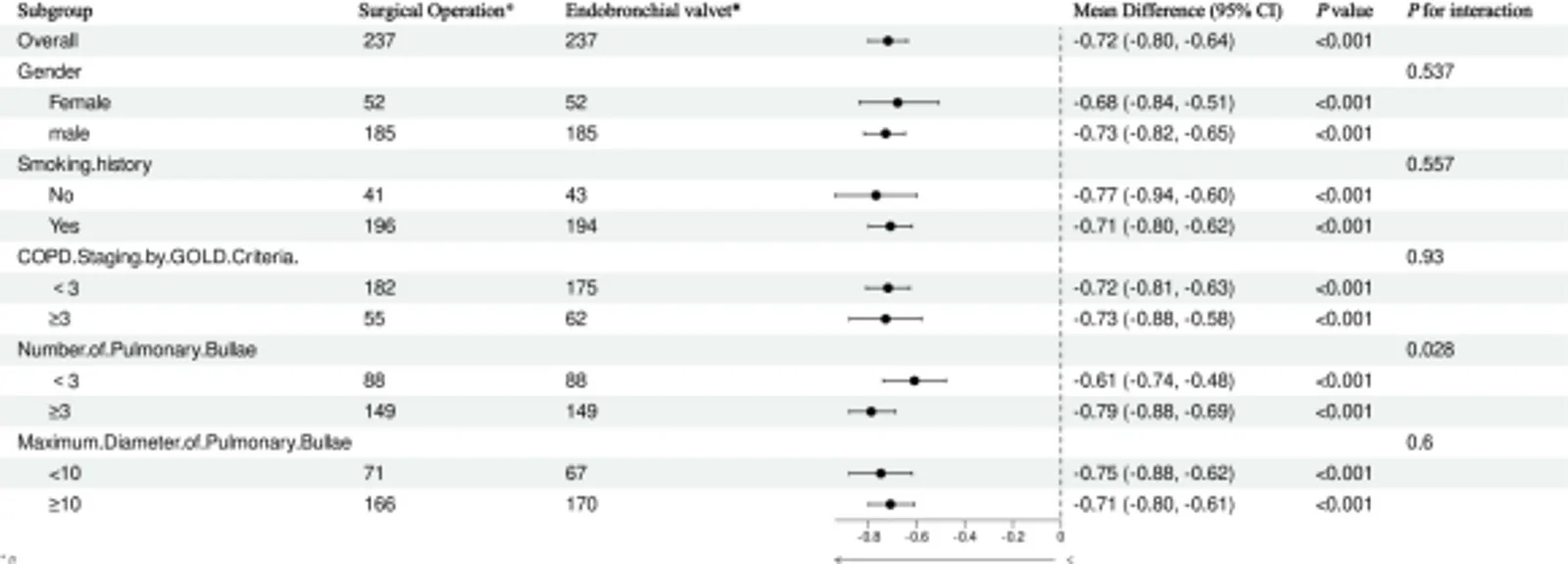
Subgroup analyses of total operation time

To further assess the generalizability of the treatment effects, we conducted subgroup analyses based on gender, smoking history, COPD staging (by GOLD criteria), number of pulmonary bullae, and maximum diameter of pulmonary bullae. Subgroup analysis of perioperative complications (Fig. 5) revealed a significant interaction based on the maximum diameter of pulmonary bullae (*P* for interaction = 0.036), indicating that endobronchial valve placement provides a more pronounced relative benefit in reducing complication risk among patients with pulmonary bullae larger than 10 cm in diameter. The subgroup analysis of total operation time (Fig. 6) revealed a significant interaction with the number of pulmonary bullae (*P* for interaction = 0.028), suggesting that EBV resulted in a significantly greater reduction in operation time for patients with a higher number of pulmonary bullae (≥ 3), with the mean difference increasing from − 0.61 to -0.79. The *P*-values for interaction were all greater than 0.05 across all predefined subgroups (Table S1-S3), indicating that the effects of the two treatment strategies on intraoperative blood loss, postoperative hospital stay, and total hospitalization cost were consistent regardless of these baseline characteristics. It should be noted that the observed differences in operative time, intraoperative blood loss, and postoperative hospital stay between EBV and SO are consistent with the inherent procedural characteristics of bronchoscopic versus surgical interventions. Therefore, these perioperative differences should not be interpreted as independent comparative advantages of one treatment modality over the other, but rather as expected consequences of the fundamentally different levels of procedural invasiveness between bronchoscopic valve placement and lung resection surgery. Therefore, these findings should be interpreted in the context of expected differences between minimally invasive endoscopic techniques and lung resection procedures. In addition, heterogeneity in surgical approaches within the SO group (VATS versus thoracotomy) may also have contributed to the observed perioperative differences.

### Comparison of secondary outcomes

The secondary outcomes of this study included all-cause readmission within one year postoperatively, re-expansion of the adjacent compressed lung tissue, and Reduction Rate of Bulla Volume on CT at 1 Year.

Table 4 presents the comparison of secondary outcomes between the two treatment groups after propensity score matching. EBV was associated with inferior outcomes compared to SO in terms of both avoiding readmission and achieving re-expansion of the adjacent lung tissue. Regarding all-cause readmission within one year, the odds of readmission were significantly higher in the EBV group (OR = 3.21, 95%CI:1.93,5.36; *p* < 0.001). Similarly, the odds of achieving radiologic re-expansion of adjacent lung tissue were significantly lower in the EBV group (OR = 0.15, 95% CI:0.06,0.36; *p* < 0.001). Analysis of the bulla volume reduction rate on CT at 1 year (Table 5) showed a significantly lower reduction in the EBV group (53.7%) compared to the SO group (82.7%), with a mean difference of -29.02% (95% CI: -31.80,-26.24; *p* < 0.001).

**Table 4:** Comparison of secondary outcomes between the Two Treatment Modalities

	Surgical operation	Endobronchial valve	Odds Ratio, OR (95% CI)	*p*-value
Readmission			3.21 (1.93–5.36)	< 0.001
Yes	24 (10%)	63 (27%)		
No	213 (90%)	174 (73%)		
Re-expansion of Adjacent Lung Tissue			0.15 (0.06–0.36)	< 0.001
Yes	231 (97%)	202 (85%)		
No	6 (2.5%)	35 (15%)		

*Abbreviations*: *CI* Confidence Interval, *OR* Odds Ratio

**Table 5:** Comparison of reduction rate of bulla volume on CT between the two treatment modalities

Character	Mean (SD)	Difference in LS Mean (95% CI)^a^	*p*-value
reduction rate of bulla volume on CT at 1 year			
Endobronchial valve -Surgical operation	53.7 (15.18) − 82.7 (15.73)	-29.02 (-31.80, -26.24)	< 0.001

^**a**^Based on an ANOVA model

*ANCOVA* Analysis of Covariance, *CI* Confidence Interval, *LS* Least Squares, *SD* Standard Deviation

To further assess the generalizability of the treatment effects, subgroup analyses were conducted based on gender, smoking history, COPD staging (by GOLD criteria), number of pulmonary bullae, and maximum diameter of pulmonary bullae. The *P*-values for interaction were all greater than 0.05 across all predefined subgroups (Table S4-S6), indicating that the differences between the two treatments in all-cause readmission within one year, radiologic re-expansion of the adjacent lung, and bulla volume reduction rate were consistent regardless of these baseline characteristics.

### Comparison of primary outcomes

The primary outcomes of this study were the FEV1% predicted at 1 year postoperatively, the change in the 6MWD at 1 year Postoperatively, and recurrence-free survival.

As shown in Table 6, the comparison of primary outcomes at 1 year postoperatively demonstrated statistically significant superiority of SO over the EBV in both lung function and exercise capacity. For the improvement in FEV1% predicted, the SO showed a greater increase from baseline (mean change: 13.6%) compared to the EBV (mean change: 3.9%). After adjusting for baseline values using an ANCOVA model, the adjusted (LS) mean improvement was 13.60% in the SO group and 3.89% in the EBV group, resulting in a significant between-group difference of -9.71% (95% CI: -10.95, -8.48; *p* < 0.001). Similarly, for the 6MWD, the SO group achieved a greater improvement from baseline (mean change: 111.7 m) than the EBV group (mean change: 59.0 m). The ANCOVA-adjusted (LS) mean improvement was 111.90 m for the SO group and 58.86 m for the EBV group, with a significant between-group difference of -53.04 m (95% CI: -64.03,-42.06; *p* < 0.001).

**Table 6:** Comparison of Primary Outcomes Between the Two Treatment Modalities

GROUP	Mean (SD)	Mean (SD)	Mean (SD)	LS Mean (95% CI)	Difference in LS Mean (95% CI)^a^	*p*-value
	Baseline	FEV1%Predicted at 1 year Postoperatively	Change from Baseline	Change from Baseline		
Surgical operation - Endobronchial valve	61.6 (16.57)- 62.5 (20.34)	75.2 (19.06) − 66.4 (22.14)	13.6 ( 8.13) − 3.9 ( 5.43)	13.60 (12.73, 14.48)-3.89 ( 3.02, 4.77)	-9.71 (-10.95, -8.48)	< 0.001
	Baseline	6-Minute Walk Distance (m) at 1 year Postoperatively	Change from Baseline	Change from Baseline		
Surgical operation - Endobronchial valve	344.3 (67.66)-340.0 (88.24)	456.0 (81.65)-399.1 (104.95)	111.7 (50.76)- 59.0 (70.30)	111.90 (104.12, 119.69)-58.86 (51.07, 66.64)	-53.04 (-64.03, -42.06)	< 0.001

^a^Based on an ANCOVA model after adjusting baseline value

*ANCOVA * Analysis of Covariance, *CI * Confidence Interval, *LS * Least Squares, *SD * Standard Deviation

**Fig. 7:**
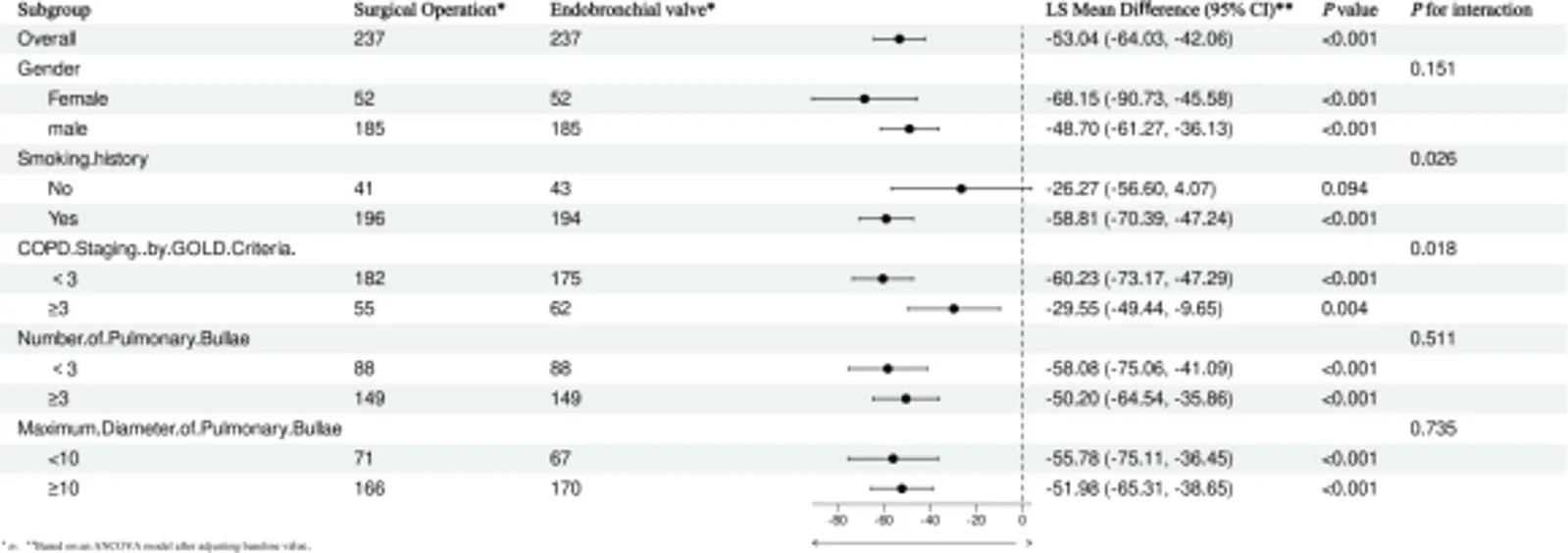
Subgroup analyses of 6MWD

Subgroup analyses (Table S7) indicated that the treatment benefits favoring surgery for FEV1% predicted were consistent across subgroups stratified by gender, smoking history, number of pulmonary bullae, and maximum diameter of pulmonary bullae (*P* for interaction > 0.05 for all). However, the analysis(Fig. 7) indicates that the treatment effect was moderated by patient baseline characteristics. The severity of chronic obstructive pulmonary disease (COPD), as staged by GOLD criteria, was a significant effect modifier for the treatment effect on the 6-minute walk distance (6MWD) (*P* for interaction = 0.018). Notably, the benefit conferred by surgery was more pronounced in patients with GOLD grade < 3(LS mean difference: -60.23 m; *P* < 0.001) compared to those with GOLD grade ≥ 3(LS mean difference: -29.55 m; *P* = 0.004). Furthermore, smoking history was also a significant effect modifier (*P* for interaction = 0.026). For patients with a history of smoking, surgical operation demonstrated a substantially greater advantage in improving 6MWD (LS mean difference: -58.81 m; *P* < 0.001). In contrast, within the subgroup of patients without a smoking history, the difference in 6MWD improvement between the two treatment modalities did not reach statistical significance (LS mean difference: -26.27 m; *P* = 0.094).

To further compare the between-group differences and temporal trends in postoperative FEV1% predicted, we performed analyses using two complementary mixed-effects models. As shown in the first model (Table 7), treating time as a continuous variable, the significant group-by-time interaction (Beta=-0.51, 95%CI: -0.60, -0.41) indicated that the rate of improvement in FEV1% predicted over time was significantly slower in the EBV group compared to the SO group. The second model (Table 8), treating time as a categorical factor, provided detailed estimates at each postoperative follow-up. Relative to the SO group, significant treatment-by-time interactions were observed at all postoperative assessments: 1 month (Beta=-9.66, 95%CI: -10.55, -8.76), 6 months (Beta=-9.62, 95%CI: -10.51, -8.73), and 12 months (Beta=-9.67, 95%CI: -10.56, -8.78). This confirms that the EBV group had significantly lower FEV1% predicted values than the SO group at every postoperative time point.

**Table 7:** the linear mixed-effects mode for FEVI% pred (Time as a Continuous Variable)

Characteristic	*N*	Beta	95% CI^a^
Group			
Surgical operation	948	Reference	Reference
Endobronchial valve	948	-4.00	-7.60, -0.39
Time	1,896	0.74	0.68, 0.81
Group * Time	1,896		
Endobronchial valve * Time	948	-0.51	-0.60, -0.41
ID.sd__(Intercept)	1,896	19.69	
Residual.sd__Observation	1,896	5.12	

^a﻿^CI = Confidence Interval,No. Obs. = 1,896; Sigma = 5.12; Log-likelihood = -6,759; AIC = 13,533; BIC = 13,572; REMLcrit = 13,519; Residual df = 1,889

**Table 8:** The linear mixed-effects mode for FEVI% pred (Time as a Categorical Variable)

Characteristic	*N*	Beta	95% CI^a^
Group			
Surgical operation	948	Reference	Reference
Endobronchial valve	948	0.84	-2.78, 4.45
Time_factor			
Preoperative	474	Reference	Reference
1Month	474	12.65	12.01, 13.28
6Months	474	13.15	12.52, 13.78
1Year	474	13.58	12.95, 14.21
Group * Time_factor	1,896		
Endobronchial valve * 1Month	237	-9.66	-10.55, -8.76
Endobronchial valve * 6 Months	237	-9.62	-10.51, -8.73
Endobronchial valve * 1Year	237	-9.67	-10.56, -8.78
ID.sd__(Intercept)	1,896	19.78	
Residual.sd__Observation	1,896	3.51	

^a^CI = Confidence Interval,No. Obs. = 1,896; Sigma = 3.51; Log-likelihood = -6,215; AIC = 12,453; BIC = 12,514; REMLcrit = 12,431; Residual df = 1,885

To further compare the between-group differences and temporal trends in postoperative 6-minute walk distance (6MWD), we performed analyses using complementary mixed-effects models. As shown in Table 9, where time was treated as a continuous variable, a significant group-by-time interaction was observed (Beta= -4.17, 95% CI: -7.09, -1.25), indicating that the rate of improvement in 6MWD over time was significantly slower in the EBV group compared to the SO group. The model treating time as a categorical factor (Table 10) provided detailed estimates at each follow-up. Significant improvements from the preoperative baseline were observed in both groups at all postoperative time points. The treatment-by-time interaction revealed that the between-group difference became statistically significant only at the 1-year assessment, where the EBV group showed 52.67 m less improvement than the SO group (95%CI: -91.51, -13.82). The differences at 1 month and 6 months were not significant.

**Table 9:** The linear mixed-effects mode for 6MWD (Time as a Continuous Variable)

Characteristic	*N*	Beta	95% CI^a^
Group			
Surgical operation	948	Reference	Reference
Endobronchial valve	948	5.09	-20.03, 30.22
Time	1,896	8.72	6.66, 10.79
Group * Time	1,896		
Endobronchial valve * Time	948	-4.17	-7.09, -1.25
ID.sd__(Intercept)	1,896	86.87	
Residual.sd__Observation	1,896	154.65	

^a^CI = Confidence Interval,No. Obs. = 1,896; Sigma = 155; Log-likelihood = -12,432; AIC = 24,876; BIC = 24,909; REMLcrit = 24,864; Residual df = 1,890

**Table 10:** the linear mixed-effects mode for 6MWD (Time as a Categorical Variable)

Characteristic	*N*	Beta	95% CI^a^
Group			
Surgical operation	948	Reference	Reference
Endobronchial valve	948	-4.24	-35.93, 27.45
Time_factor			
Preoperative	474	Reference	Reference
1Month	474	35.16	7.69, 62.63
6Months	474	90.80	63.33, 118.27
1Year	474	111.71	84.25, 139.18
Group * Time_factor	1,896		
Endobronchial valve * 1Month	237	2.01	-36.84, 40.85
Endobronchial valve * 6 Months	237	8.75	-30.10, 47.59
Endobronchial valve * 1Year	237	-52.67	-91.51, -13.82
ID.sd__(Intercept)	1,896	87.80	
Residual.sd__Observation	1,896	152.56	

^a^CI = Confidence Interval,No. Obs. = 1,896; Sigma = 153; Log-likelihood = -12,392; AIC = 24,804; BIC = 24,859; REMLcrit = 24,784; Residual df = 1,886

**Fig. 8:**
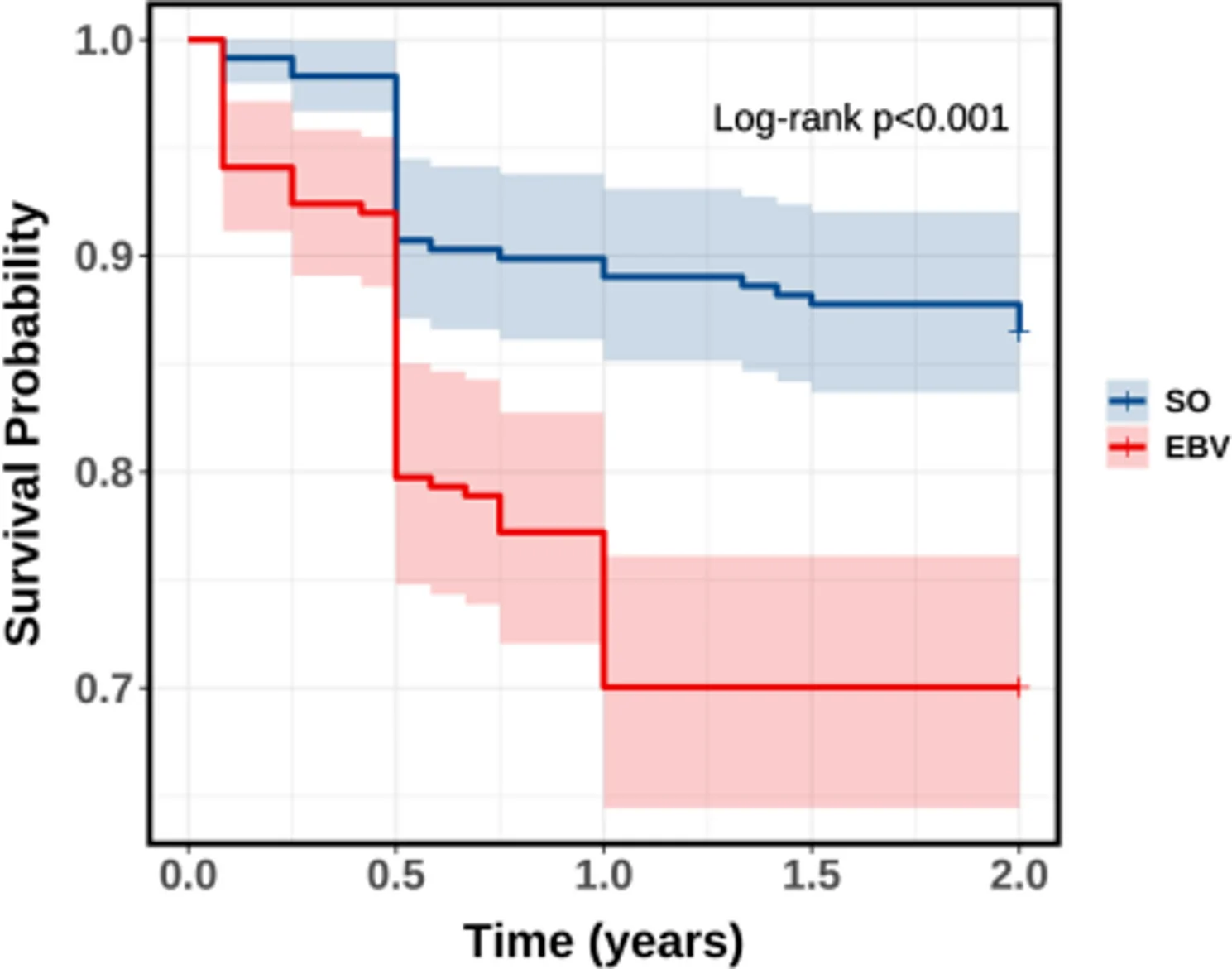
Recurrence-free survival curve

Kaplan-Meier survival analysis (Table 11; Fig. 8) showed that in the overall population (*n* = 474), the 1-year recurrence-free survival rate was 79.5% (95% CI: 76.0%, 83.3%), and the 2-year recurrence-free survival rate was 78.3% (95% CI: 74.6%, 82.1%). The 1 and 2-year recurrence-free survival rates were 89.0% (95% CI: 85.1%, 93.1%) and 86.5% (95% CI: 82.3%, 91.0%), respectively, in the SO group, compared to 70.0% (95% CI: 64.4%, 76.1%) for both time points in the EBV group. Between-group comparison revealed a statistically significant difference in recurrence-free survival (log-rank test, *p* < 0.001) Table 12.

**Table 11:** Kaplan-meier Estimates for Survival Rates (95% CI)

Characteristic	*N*	Event *N*	1-year	2-year	*p*-value^a^
Overall	474	103	79.5% (76.0%, 83.3%)	78.3% (74.6%, 82.1%)	
Group	474	103			< 0.001
Surgical operation			89.0% (85.1%, 93.1%)	86.5% (82.3%, 91.0%)	
Endobronchial valve			70.0% (64.4%, 76.1%)	70.0% (64.4%, 76.1%)	

^a^Log-rank test

**Table 12:** Cox Regression for the association between treatment modality and recurrence-free survival

Characteristic	*N*	Event *N*	HR	95% CI	*p*-value
Group					
Surgical operation	237	32	Reference	Reference	
Endobronchial valve	237	71	2.48	1.63, 3.77	< 0.001

*Abbreviations*: *CI * Confidence Interval, *HR* Hazard Ratio

**Fig. 9:**
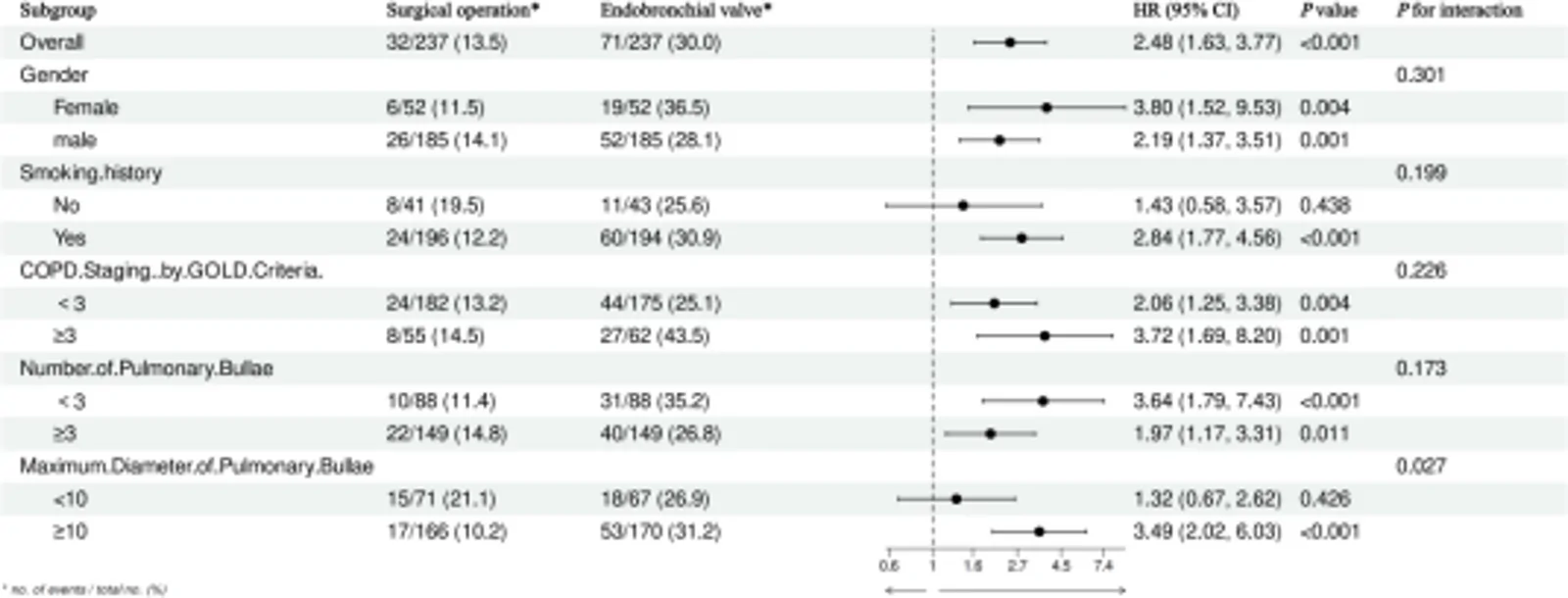
Subgroup analyses of the treatment effect on recurrence-free survival

In the Cox proportional hazards model assessing the association between treatment modality and recurrence-free survival, patients receiving EBV exhibited a significantly higher risk of recurrence compared to those undergoing SO (HR 2.48, 95%CI 1.63, 3.77; *p* < 0.001). Subgroup analyses were conducted to assess the consistency of the treatment effect on recurrence-free survival (Fig. 9). The increased recurrence risk associated with EBV was consistently observed across most predefined subgroups, including gender, smoking history, COPD severity, and number of bullae (all *P* for interaction > 0.05). However, a significant interaction was found with the maximum diameter of pulmonary bullae (*P* for interaction = 0.027). Notably, for bullae with a maximum diameter < 10 cm, there was no significant difference in recurrence risk between the two treatments (HR 1.32, 95% CI 0.67,2.62). In contrast, for giant bullae (≥ 10 cm), the risk of recurrence was significantly higher in the EBV group compared to the surgical group (HR 3.49, 95% CI 2.02,6.03).

## Discussion

Our comparative analysis reveals a stark trade-off in the management of refractory giant bullae. Surgical bullectomy provides a durable and cost-effective solution, demonstrating superiority in long-term functional improvement and recurrence prevention, albeit at the cost of higher initial invasiveness and a slower postoperative recovery. In contrast, bronchoscopic valve placement serves as a less invasive option with a quicker recovery profile, but this is offset by its compromised long-term efficacy, increased recurrence rates, and higher direct healthcare expenditures. The paradoxical finding of higher costs for the minimally invasive approach can be largely attributed to the substantial expense of the endobronchial valves. As high-value, single-use, imported devices, they constitute the principal cost-driving factor. With individual valve units priced at several thousand dollars and treatment often requiring the implantation of multiple valves, the total procedural cost escalates significantly [19]. Conversely, the cumulative cost of surgical staplers and other consumables used in a bullectomy, though considerable, generally remains below that of a multi-valve bronchoscopic procedure.

In the present study, patients in the surgical operation (SO) group demonstrated greater improvements in both pulmonary function and exercise capacity at 1 year, with between-group differences of + 9.71% in FEV1% predicted and + 53.04 m in 6MWD. These findings are consistent with the underlying physiological rationale that surgical resection enables more complete removal of non-functional bullous tissue, thereby reducing the compressive effect on adjacent lung parenchyma and promoting its re-expansion. Our results are broadly consistent with previous studies evaluating lung volume reduction strategies. In particular, the randomized controlled trial by Lu et al. [20] reported that both surgical and endobronchial approaches can improve functional outcomes, although the magnitude and durability of benefit may differ between modalities. Compared with that prospective study, our findings suggest a more pronounced and sustained improvement in the surgical group, particularly at longer follow-up time points. This difference may be explained by variations in patient selection, disease characteristics, and study design. In our cohort, a substantial proportion of patients presented with giant bullae, in which complete anatomical resection may confer a more definitive mechanical advantage. In contrast, bronchoscopic approaches such as EBV rely on functional volume reduction and may be more sensitive to factors such as collateral ventilation and disease heterogeneity. Additionally, longitudinal analysis using mixed-effects models in our study demonstrated that, although both groups experienced sustained postoperative improvement, the SO group maintained consistently higher FEV1% predicted across all follow-up time points. This temporal pattern further supports the notion that surgical intervention may provide a more durable functional benefit within the first postoperative year, although these findings should be interpreted in light of differences in study design and potential residual confounding.

The longitudinal trajectory of functional recovery, as measured by the 6-minute walk distance (6MWD), demonstrated a complex and divergent pattern between the two treatment strategies. While a significant time effect confirmed functional improvement in both cohorts, the critical finding was a significant treatment-by-time interaction. When modelled continuously, the rate of functional gain was significantly slower in the endobronchial valve group (EBV*Timeβ=-4.17). Analysis of categorical time points revealed that the between-group difference, negligible at 1 and 6 months, evolved into a substantial and statistically significant disadvantage for the EBV group by 1 year (β= -52.67 m). This pattern suggests that surgical patients, after the initial perioperative burden, embark on a steeper and more sustained recovery trajectory, culminating in superior long-term functional capacity. Conversely, the recovery curve for the EBV group, while showing early promise, plateaued at a lower level. This divergence highlights a fundamental difference: surgery provides a greater long-term functional reserve, whereas the minimally invasive approach may offer quicker initial gains but with a lower efficacy ceiling for sustained functional improvement.

The most striking contrast between the two strategies was observed in the long-term outcome of recurrence-free survival. Kaplan-Meier analysis and Cox regression models unequivocally demonstrated the decisive advantage of surgery in providing durable bulla control. With a hazard ratio of approximately 2.5, the endobronchial valve group exhibited a more than twofold increase in the risk of recurrence. This considerable disparity underscores a fundamental limitation of EBV, its inability to achieve the same degree of long-term therapeutic durability as surgery.

However, the superior efficacy of surgery came with notable trade-offs. Persistent air leak represented the most frequent postoperative complication in the surgical cohort. This finding is consistent with previous reports of bullectomy and lung volume reduction procedures, where postoperative air leakage remains a major determinant of prolonged hospitalization and resource utilization. Although most cases in our cohort resolved with conservative chest tube management, the occurrence of persistent air leak highlights the inherent invasiveness of surgical intervention and should be considered when selecting treatment strategies, particularly in patients with limited pulmonary reserve. Our objective data indicated that the SO group experienced longer operative times, greater intraoperative blood loss, delayed first ambulation, and prolonged postoperative hospital stays. In contrast, the EBV group exemplified the core advantages of a minimally invasive approach, with minimal blood loss and prompt postoperative mobilization and recovery. Its principal limitations lie in its modest efficacy and higher long-term recurrence risk. This finding may be attributable to the limited sample size of the present study, and future larger-scale investigations are warranted to precisely evaluate differences in perioperative complications between these two modalities.

Based on our subgroup analyses, key patient characteristics significantly moderated therapeutic responses, clarifying which patients derive the greatest benefit from each intervention. First, COPD severity modified functional outcomes. While surgery significantly improved 6MWD across all stages, the benefit was substantially greater in patients with GOLD grade < 3 (difference:−60.23 m) than in those with grade ≥ 3 (difference:−29.55 m). This suggests that in advanced diffuse emphysema, overall functional capacity may be limited more by the underlying parenchymal disease than by the bulla itself, thereby attenuating the additional gains achievable from surgical resection. Second, smoking history emerged as an important modifier of functional recovery. Patients with a smoking history derived markedly greater 6MWD improvement from surgery (difference:-58.81 m) compared to non-smokers (difference: -26.27 m), in whom the between-group difference was not statistically significant. This may reflect distinct disease phenotypes, inflammatory profiles, or physiological reserve between these subgroups. Most critically, bulla size was a decisive modifier of long‑term disease control. For bullae < 10 cm in diameter, recurrence risk did not differ significantly between treatments (HR 1.32, 95% CI 0.67–2.62). In contrast, for giant bullae (≥ 10 cm), the risk of recurrence was more than threefold higher with EBV (HR 3.49, 95% CI 2.02–6.03). This indicates that the anatomical completeness of surgical resection provides a decisive and durable advantage for large bullous lesions, an advantage that bronchoscopic intervention may not reliably replicate.

Taken together, these findings support a refined, multidimensional decision framework. Surgical bullectomy is best suited for patients with adequate physiological reserve (GOLD grade < 3), particularly those with a smoking history and those with giant bullae (≥ 10 cm), in whom it offers superior and durable disease control. Endobronchial valve placement represents a viable minimally invasive alternative for higher risk patients, especially those with severe COPD (GOLD ≥ 3), non-smokers, or those with smaller bullae (< 10 cm), who may prioritize reduced procedural trauma and faster recovery over maximal long-term efficacy.

Postoperative chest tube management was performed according to standardized institutional protocols, including assessment of air leak status, drainage volume, and radiographic lung re-expansion. These practices are consistent with contemporary thoracic surgical management principles and may have contributed to the relatively low incidence of severe postoperative complications observed in the present study. Several limitations of this study should be acknowledged. First, the retrospective and non-randomized design inherently introduces the risk of selection bias and unmeasured confounding. Although propensity score matching and multivariable analyses were performed to balance observed baseline characteristics, treatment allocation was based on real-world clinical decision-making. As such, residual confounding by indication cannot be fully excluded, as factors such as clinician judgment, patient preference, and subtle differences in physiological reserve may have influenced treatment selection. Second, the characterization of baseline clinical profiles was limited by the availability of retrospectively collected data. Several important covariates that may affect both treatment selection and outcomes including comorbidity burden, frailty status, diffusion capacity (DLCO), detailed emphysema distribution, prior pneumothorax, and nutritional status were not consistently recorded and therefore could not be incorporated into the propensity score model. In addition, collateral ventilation status, a key determinant of EBV efficacy, was only assessed in the EBV group and could not be included in baseline adjustment. Third, the heterogeneity of surgical approaches within the SO group (VATS versus open thoracotomy) was not fully captured due to incomplete documentation. This may have influenced perioperative outcomes such as operative time, blood loss, and length of hospital stay. This limitation is particularly relevant when interpreting perioperative outcomes, including operative time, intraoperative blood loss, postoperative complications, and length of hospital stay, all of which are known to be strongly influenced by the surgical approach itself. Compared with VATS, open thoracotomy is generally associated with greater surgical trauma, longer recovery time, and higher perioperative morbidity. Therefore, the inability to fully stratify or adjust for the proportion of VATS versus thoracotomy cases within the surgical group may have introduced additional heterogeneity into the perioperative comparisons between surgical operation and endobronchial valve treatment. As a result, part of the observed differences in perioperative outcomes may reflect variation in surgical approach rather than the intrinsic differences between surgical and bronchoscopic treatment strategies alone. Fourth, several outcome definitions and measurement methods have inherent limitations. Recurrence was defined as a composite endpoint including radiologic progression, pneumothorax recurrence, or the need for reintervention, which may encompass heterogeneous clinical scenarios. Perioperative complications were identified from retrospective clinical records without standardized severity grading, which may introduce variability in reporting. In addition, radiologic outcomes were assessed using semi-quantitative methods, and bulla size reduction was estimated using two-dimensional measurements rather than true volumetric analysis, potentially limiting measurement precision. Furthermore, the imaging-based outcome measures used in this study have not been formally validated as standardized quantitative endpoints for bullous lung disease. Both the semi-quantitative assessment of adjacent lung re-expansion and the two-dimensional surrogate measurement of bulla reduction may be subject to observer variability and limited reproducibility. In particular, the use of simplified imaging metrics may not fully capture the complex three-dimensional morphological changes of bullous lesions following treatment. Therefore, the radiologic findings should be interpreted cautiously, and the observed between-group differences in imaging outcomes may be influenced by limitations in measurement methodology. Future studies incorporating standardized three-dimensional volumetric analysis and validated imaging assessment protocols are warranted to improve the robustness and precision of radiologic outcome evaluation. Fifth, the follow-up duration of two years limits the assessment of long-term durability of treatment effects. Furthermore, the economic analysis was restricted to direct medical costs and did not account for indirect or societal costs. Finally, the generalizability of our findings may be limited, as the study population consisted exclusively of Chinese patients. Differences in genetic background, environmental exposure, and healthcare systems may affect the applicability of these results to other populations. Importantly, several clinically relevant covariates that likely influenced treatment selection and prognosis were not consistently available in the retrospective dataset and therefore could not be incorporated into the propensity score model. Accordingly, the results of the present study should be interpreted primarily as hypothesis-generating rather than definitive comparative evidence. While the observed associations remained consistent after propensity score matching, the possibility that unmeasured baseline differences contributed to the observed treatment effects cannot be excluded. Therefore, our findings should be viewed as supportive evidence for future prospective investigations rather than conclusive proof of treatment superiority.

Future research should focus on prospective, multicenter, and international studies with more comprehensive data collection, including detailed physiological, radiological, and functional parameters. The use of standardized outcome definitions and advanced imaging quantification methods will further improve the robustness and clinical applicability of comparative effectiveness research in refractory bullous lung disease.

## Conclusion

In summary, SO and EBV represent two effective yet distinctly different therapeutic strategies for refractory bullae. In this propensity score-matched retrospective cohort, surgical operation was associated with greater improvements in pulmonary function, exercise capacity, and recurrence-free survival compared with endobronchial valve treatment. However, because of the observational study design and the potential for residual confounding, these findings should be interpreted cautiously and should not be considered definitive evidence of treatment superiority. Prospective multicenter studies and randomized trials are required to validate these observations. Therefore, the therapeutic decision should not follow a one-size-fits-all approach but rather be guided by an integrated consideration of efficacy, risks, costs, and key patient characteristics such as COPD staging and baseline pulmonary function to achieve truly personalized patient treatment.

## Supplementary Information

Supplementary Material 1.

## Data Availability

The datasets used and/or analysed during the current study are available from the corresponding author on reasonable request.
