# Supplementary material for: The efficacy-invasiveness trade-off: a retrospective cohort comparison of surgical operation and endobronchial valves for refractory lung bullae disease
Source: BMC Pulm Med. 2026 Jul 10;26:448. doi: 10.1186/s12890-026-04488-5 (PMC13637419; doi:10.1186/s12890-026-04488-5)
Supplement: Supplementary file 1 [file 12890_2026_4488_MOESM1_ESM.docx]

**Table S1 Subgroup Analysis for Total.Intraoperative.Blood.Loss. (ml)**

| **Subgroup** | **SO** | **EBV** | **Mean Difference (95% CI)^1^** | **P value** | **P for interaction** |
| --- | --- | --- | --- | --- | --- |
| Overall | 237 | 237 | -48.00 (-54.11, -41.90) | <0.001 |  |
| Gender |  |  |  |  | 0.632 |
| Female | 52 | 52 | -50.83 (-61.33, -40.33) | <0.001 |  |
| male | 185 | 185 | -47.21 (-54.47, -39.95) | <0.001 |  |
| Smoking.history |  |  |  |  | 0.541 |
| No | 41 | 43 | -43.87 (-54.23, -33.51) | <0.001 |  |
| Yes | 196 | 194 | -48.87 (-55.95, -41.78) | <0.001 |  |
| COPD.Staging..by.GOLD.Criteria. |  |  |  |  | 0.132 |
| ˂3 | 182 | 175 | -45.49 (-52.44, -38.53) | <0.001 |  |
| ≥3 | 55 | 62 | -56.37 (-69.02, -43.72) | <0.001 |  |
| Number.of.Pulmonary.Bullae |  |  |  |  | 0.398 |
| ˂3 | 88 | 88 | -44.57 (-53.21, -35.93) | <0.001 |  |
| ≥3 | 149 | 149 | -50.03 (-58.31, -41.76) | <0.001 |  |
| Maximum.Diameter.of.Pulmonary.Bullae |  |  |  |  | 0.137 |
| ˂10 | 71 | 67 | -40.88 (-51.28, -30.48) | <0.001 |  |
| ≥10 | 166 | 170 | -51.06 (-58.51, -43.61) | <0.001 |  |
| ^1^Based on an ANOVA model. | | | | | |

**Table S2 Subgroup Analysis for Postoperative.Hospital.Stay.(d).**

| **Subgroup** | **SO** | **EBV** | **Mean Difference (95% CI)^1^** | **P value** | **P for interaction** |
| --- | --- | --- | --- | --- | --- |
| Overall | 237 | 237 | -7.61 (-8.33, -6.88) | <0.001 |  |
| Gender |  |  |  |  | 0.157 |
| Female | 52 | 52 | -8.60 (-10.37, -6.82) | <0.001 |  |
| male | 185 | 185 | -7.33 (-8.11, -6.55) | <0.001 |  |
| Smoking.history |  |  |  |  | 0.587 |
| No | 41 | 43 | -7.17 (-8.78, -5.56) | <0.001 |  |
| Yes | 196 | 194 | -7.70 (-8.51, -6.89) | <0.001 |  |
| COPD.Staging..by.GOLD.Criteria. |  |  |  |  | 0.215 |
| ˂3 | 182 | 175 | -7.38 (-8.15, -6.60) | <0.001 |  |
| ≥3 | 55 | 62 | -8.44 (-10.18, -6.70) | <0.001 |  |
| Number.of.Pulmonary.Bullae |  |  |  |  | 0.054 |
| ˂3 | 88 | 88 | -6.68 (-7.82, -5.55) | <0.001 |  |
| ≥3 | 149 | 149 | -8.15 (-9.09, -7.22) | <0.001 |  |
| Maximum.Diameter.of.Pulmonary.Bullae |  |  |  |  | 0.749 |
| ˂10 | 71 | 67 | -7.42 (-8.93, -5.91) | <0.001 |  |
| ≥10 | 166 | 170 | -7.68 (-8.50, -6.86) | <0.001 |  |
| ^1^Based on an ANOVA model. | | | | | |

**Table S3 Subgroup Analysis for Total.Hospitalization.Cost.(RMB)**

| **Subgroup** | **SO** | **EBV** | **Mean Difference (95% CI)^1^** | **P value** | **P for interaction** |
| --- | --- | --- | --- | --- | --- |
| Overall | 237 | 237 | 104502.21 (99719.11, 109285.30) | <0.001 |  |
| Gender |  |  |  |  | 0.365 |
| Female | 52 | 52 | 100326.03 (90490.73, 110161.32) | <0.001 |  |
| male | 185 | 185 | 105676.05 (100206.83, 111145.28) | <0.001 |  |
| Smoking.history |  |  |  |  | 0.384 |
| No | 41 | 43 | 109073.72 (96700.97, 121446.48) | <0.001 |  |
| Yes | 196 | 194 | 103495.48 (98321.23, 108669.73) | <0.001 |  |
| COPD.Staging..by.GOLD.Criteria. |  |  |  |  | 0.212 |
| ˂3 | 182 | 175 | 106280.34 (101167.74, 111392.94) | <0.001 |  |
| ≥3 | 55 | 62 | 99200.21 (87651.06, 110749.36) | <0.001 |  |
| Number.of.Pulmonary.Bullae |  |  |  |  | 0.478 |
| ˂3 | 88 | 88 | 102244.64 (93393.19, 111096.09) | <0.001 |  |
| ≥3 | 149 | 149 | 105835.54 (100289.46, 111381.61) | <0.001 |  |
| Maximum.Diameter.of.Pulmonary.Bullae |  |  |  |  | 0.595 |
| ˂10 | 71 | 67 | 102441.47 (94251.89, 110631.04) | <0.001 |  |
| ≥10 | 166 | 170 | 105303.15 (99443.79, 111162.51) | <0.001 |  |
| ^1^Based on an ANOVA model. | | | | | |

**Table S4 Subgroup Analysis for Readmission**

| **Subgroup** | **SO^1^** | **EBV^1^** | **Crude OR**  **(95% CI)** | **P value** | **P for interaction** |
| --- | --- | --- | --- | --- | --- |
| Overall | 24/237 (10.1) | 63/237 (26.6) | 3.21 (1.93-5.36) | <0.001 |  |
| Gender |  |  |  |  | 0.102 |
| Female | 2/52 (3.8) | 15/52 (28.8) | 10.14 (2.18-47.06) | 0.003 |  |
| male | 22/185 (11.9) | 48/185 (25.9) | 2.60 (1.49-4.51) | 0.001 |  |
| Smoking.history |  |  |  |  | 0.956 |
| No | 5/41 (12.2) | 13/43 (30.2) | 3.12 (1.00-9.75) | 0.050 |  |
| Yes | 19/196 (9.7) | 50/194 (25.8) | 3.23 (1.83-5.73) | <0.001 |  |
| COPD.Staging..by.GOLD.Criteria. |  |  |  |  | 0.474 |
| ˂3 | 15/182 (8.2) | 43/175 (24.6) | 3.63 (1.93-6.81) | <0.001 |  |
| ≥3 | 9/55 (16.4) | 20/62 (32.3) | 2.43 (1.00-5.93) | 0.050 |  |
| Number.of.Pulmonary.Bullae |  |  |  |  | 0.100 |
| ˂3 | 7/88 (8.0) | 35/88 (39.8) | 7.64 (3.16-18.47) | <0.001 |  |
| ≥3 | 17/149 (11.4) | 28/149 (18.8) | 1.80 (0.94-3.45) | 0.078 |  |
| Maximum.Diameter.of.Pulmonary.Bullae |  |  |  |  | 0.512 |
| ˂10 | 8/71 (11.3) | 16/67 (23.9) | 2.47 (0.98-6.23) | 0.055 |  |
| ≥10 | 16/166 (9.6) | 47/170 (27.6) | 3.58 (1.94-6.63) | <0.001 |  |
| ^1^no. of events / total no. (%) | | | | | |

**Table S5 Subgroup Analysis for Re-expansion of Adjacent Lung Tissue**

| **Subgroup** | **SO^1^** | **EBV^1^** | **Crude OR**  **(95% CI)** | **P value** | **P for interaction** |
| --- | --- | --- | --- | --- | --- |
| Overall | 231/237 (97.5) | 202/237 (85.2) | 0.15 (0.06-0.36) | <0.001 |  |
| Gender |  |  |  |  | 0.873 |
| Female | 50/52 (96.2) | 40/52 (76.9) | 0.13 (0.03-0.63) | 0.011 |  |
| male | 181/185 (97.8) | 162/185 (87.6) | 0.16 (0.05-0.46) | 0.001 |  |
| Smoking.history |  |  |  |  | 0.229 |
| No | 39/41 (95.1) | 38/43 (88.4) | 0.39 (0.07-2.13) | 0.277 |  |
| Yes | 192/196 (98.0) | 164/194 (84.5) | 0.11 (0.04-0.33) | <0.001 |  |
| COPD.Staging..by.GOLD.Criteria. |  |  |  |  | 0.922 |
| ˂3 | 178/182 (97.8) | 153/175 (87.4) | 0.16 (0.05-0.46) | 0.001 |  |
| ≥3 | 53/55 (96.4) | 49/62 (79.0) | 0.14 (0.03-0.66) | 0.013 |  |
| Number.of.Pulmonary.Bullae |  |  |  |  | 0.294 |
| ˂3 | 86/88 (97.7) | 69/88 (78.4) | 0.08 (0.02-0.38) | 0.001 |  |
| ≥3 | 145/149 (97.3) | 133/149 (89.3) | 0.23 (0.07-0.70) | 0.010 |  |
| Maximum.Diameter.of.Pulmonary.Bullae |  |  |  |  | 0.737 |
| ˂10 | 69/71 (97.2) | 54/67 (80.6) | 0.12 (0.03-0.56) | 0.007 |  |
| ≥10 | 162/166 (97.6) | 148/170 (87.1) | 0.17 (0.06-0.49) | 0.001 |  |
| ^1^no. of events / total no. (%) | | | | | |

**Table S6 Subgroup Analysis for Reduction Rate of Bulla Volume on CT at 1 Year**

| **Subgroup** | **SO** | **EBV** | **Mean Difference (95% CI)^1^** | **P value** | **P for interaction** |
| --- | --- | --- | --- | --- | --- |
| Overall | 237 | 237 | -29.02 (-31.80, -26.24) | <0.001 |  |
| Gender |  |  |  |  | 0.041 |
| Female | 52 | 52 | -34.50 (-41.54, -27.46) | <0.001 |  |
| male | 185 | 185 | -27.48 (-30.44, -24.53) | <0.001 |  |
| Smoking.history |  |  |  |  | 0.47 |
| No | 41 | 43 | -31.24 (-38.30, -24.18) | <0.001 |  |
| Yes | 196 | 194 | -28.55 (-31.58, -25.53) | <0.001 |  |
| COPD.Staging..by.GOLD.Criteria. |  |  |  |  | 0.409 |
| ˂3 | 182 | 175 | -28.28 (-31.28, -25.29) | <0.001 |  |
| ≥3 | 55 | 62 | -31.01 (-37.63, -24.39) | <0.001 |  |
| Number.of.Pulmonary.Bullae |  |  |  |  | 0.133 |
| ˂3 | 88 | 88 | -31.80 (-36.76, -26.83) | <0.001 |  |
| ≥3 | 149 | 149 | -27.38 (-30.69, -24.07) | <0.001 |  |
| Maximum.Diameter.of.Pulmonary.Bullae |  |  |  |  | 0.37 |
| ˂10 | 71 | 67 | -27.01 (-32.06, -21.95) | <0.001 |  |
| ≥10 | 166 | 170 | -29.82 (-33.15, -26.48) | <0.001 |  |
| ^1^Based on an ANOVA model. | | | | | |

**Table S7 Subgroup Analysis for FEV1% predicted at 1 year postoperatively**

| **Subgroup** | **SO** | **EBV** | **Mean Difference (95% CI)^1^** | **P value** | **P for interaction** |
| --- | --- | --- | --- | --- | --- |
| Overall | 237 | 237 | -9.71 (-10.95, -8.48) | <0.001 |  |
| Gender |  |  |  |  | 0.406 |
| Female | 52 | 52 | -11.24 (-13.35, -9.12) | <0.001 |  |
| male | 185 | 185 | -9.45 (-10.91, -7.98) | <0.001 |  |
| Smoking.history |  |  |  |  | 0.199 |
| No | 41 | 43 | -11.40 (-13.74, -9.07) | <0.001 |  |
| Yes | 196 | 194 | -9.34 (-10.76, -7.93) | <0.001 |  |
| COPD.Staging..by.GOLD.Criteria. |  |  |  |  | 0.581 |
| ˂3 | 182 | 175 | -9.83 (-11.31, -8.34) | <0.001 |  |
| ≥3 | 55 | 62 | -9.25 (-11.30, -7.20) | <0.001 |  |
| Number.of.Pulmonary.Bullae |  |  |  |  | 0.202 |
| ˂3 | 88 | 88 | -8.69 (-10.46, -6.93) | <0.001 |  |
| ≥3 | 149 | 149 | -10.35 (-12.05, -8.66) | <0.001 |  |
| Maximum.Diameter.of.Pulmonary.Bullae |  |  |  |  | 0.086 |
| ˂10 | 71 | 67 | -11.04 (-13.95, -8.12) | <0.001 |  |
| ≥10 | 166 | 170 | -8.97 (-10.23, -7.72) | <0.001 |  |
| ^1^Based on an ANCOVA model after adjusting baseline value. | | | | | |
